# On the limits of inferring biophysical parameters of RBP-RNA interactions from in vitro RNA Bind’n Seq data

**DOI:** 10.12688/f1000research.135164.1

**Published:** 2023-06-26

**Authors:** Niels Schlusser, Mihaela Zavolan

**Affiliations:** 1 Biozentrum, Universitat Basel, Basel, Basel-Stadt, 4056, Switzerland

**Keywords:** Systems biology, bioinformatics, computational biology, machine learning, maximum entropy method, Bayesian statistics, RNA binding proteins, RNA Bind'n'Seq

## Abstract

We develop a thermodynamic model describing the binding of RNA binding proteins (RBP) to oligomers in vitro. We apply expectation-maximization to infer the specificity of RBPs, represented as position-specific weight matrices (PWMs), by maximizing the likelihood of RNA Bind’n Seq data from the ENCODE project. We demonstrate that the model can reproduce known specificities for well-studied proteins and that in some cases we predict novel, longer binding motifs. However, the model does not recover all the motifs that are in principle known, indicating that the data is not well explained by a single underlying biophysical model. Our code is publicly available.

## 1. Introduction

RNA-binding proteins (RBPs) interact with RNAs at every step of their life cycle. Their modular structure, usually an assortment of RNA-binding domains, underlies their ability to interact with both RNAs and proteins, and couple various layers of gene expression. ^1^ While ∼2500 RBPs are currently known, most remain to be functionally characterized. A first step in this process is to determine the interaction partners and the sequence/structure specificity of the RBP. Many RBPs recognize their targets in a sequence-specific manner, although the accessibility of binding sites within the targets also plays a role. ^2^ The sequence specificity is usually represented in a position weight matrix (PWM), which specifies the probability of finding each of the four nucleotides at each position in the RBP binding site. This is an obvious simplification, as dependencies between positions in the binding site likely occur. However, training more complex models requires substantially more data, which are often not available. Moreover, the improvement in binding site predictability by more complex models is modest, at least in the case of other nucleic acid binding proteins, transcription factors. ^3^ With the realization that the presence of a canonical RNA-binding domains is not necessary for the ability of a protein to bind RNAs ^4^ came a pressing need to determine the determinants of RNA-RBPs interactions and the sequence/structure specificity of the proteins newly found to interact with RNAs.

The past two decades have seen the development and broad application of experimental methods for RBP target identification. They include *in vivo* high-throughput approaches such as HITS-CLIP, PAR-CLIP, iCLIP and eCLIP (reviewed in Ref. 5), and more recently-developed *in vitro* approaches such as RNA Bind’n Seq. ^6^ While the CLIP methods rely on the sequencing of RNAs that interact and can therefore be crosslinked to RBPs *in vivo*, RNA Bind’n Seq relies on the affinity-dependent interaction of RBPs with random RNAs *in vitro.* The oligonucleotides whose interaction with the RBP (or domain thereof) of interest are computationally analyzed to identify short sequence motifs that are enriched in the affinity-selected pool of RNAs. So far, analyses of such data involved the identification of enriched k-mers (short oligonucleotide sequences of a specified length, k ), and then a greedy alignment procedure yielded PWM representations of the RBP binding motifs. This left open the question of whether the derived PWMs accurately predicted the interaction energies of RBPs with their binding sites. In contrast, the aim of our work was to develop a biophysics-anchored method to directly infer the PWMs from RNA Bind’n Seq data. Our paper is organized as follows: Sec. 2 explains how we derive our thermodynamical model. We comment on the practical implementation of this model in Sec. 3, where we also explain how we account for sequence composition biases in the pool of oligomers. Results for different RBPs are presented in Sec. 4, where we also comment on the accuracy of the results obtained from this type of data for different RBPs. Concluding remarks are given in Sec. 5, and a list of publicly available data sets that we analyzed is provided in Sec. 6.

## 2. Model

Our model is an adaptation of a Bayesian, thermodynamic model that was constructed to infer di-nucleotide weight tensors from SELEX data. ^7^ In the following, we derive the log-likelihood of Bind’N Seq data given the PWM for the RBP of interest, which will be inferred by expectation-maximization as described in Sec. 3.

We assume that an RBP binds an oligomer over a binding site s of length $L_{w}$ and that the likelihood of the binding taking place, according to Boltzmann’s law, goes as $\propto \exp\left(\sum_{i=1}^{L_{w}}E_{i}^{s_{i}}\right)\equiv e^{E\left(s\right)}$ , where $s_{i}$ is the nucleotide at position i , so $s_{i}\in \left\{A,C,G,T\right\}$ . Therefore, each element of the position weight matrix (PWM) M can be identified with $m_{i}^{\alpha }\equiv \exp\left(E_{i}^{\alpha }\right)$ , with their columns being normalized as $\sum_{\alpha }m_{i}^{\alpha }=1\;\forall \;i=1,\ldots ,L_{w}$ .

Additionally, we account for the fact that there are genuinely two different ways of binding, sequence-specific binding as described by the PWM, and unspecific binding to RNAs with a probability $\exp\left(E_{0}\right)$ . Combining these two possibilities, we arrive at the probability for an RBP binding to a site s

$$P_{b}\left(s|c,M,E_{0}\right)=\frac{c\left(e^{E\left(s\right)}+e^{E_{0}}\right)}{1+c\left(e^{E\left(s\right)}+e^{E_{0}}\right)},$$ (2.1)

where the 1 in the denominator represents the (constant) chance of an RBP being unbound which is set to 1 by normalizing the protein concentrations c , accordingly. Note that $E_{0}\leq 0$ needs to be satisfied since $e^{E_{0}}$ is a probability which, in turn, needs to satisfy $e^{E_{0}}\leq 1$ . Exploiting the fact that the binding of RBPs to oligomers is not saturated, i.e. c≪1 , we can linearize (2.1)

$$P_{b}\left(s|c,M,E_{0}\right)\approx c\left(e^{E\left(s\right)}+e^{E_{0}}\right).$$ (2.2)

Consequently, the chance of an RBP being bound somewhere on a longer oligomer S with $L_{S}\geq L_{w}$ is

$$P_{b}\left(S|c,M,E_{0}\right)=\sum_{s\in S}P_{b}\left(s|c,M,E_{0}\right)\approx c\left(e^{E\left(S\right)}+\left(L_{S}-L_{w}+1\right)e^{E_{0}}\right),$$ (2.3)

where $e^{E\left(S\right)}\equiv \sum_{s\in S}e^{E\left(s\right)}$ and a sum over all possible $L_{w}$ -mers s in S . The probability of each read S in the pool of oligomers that are washed over the RBP is

$$P_{\mathrm{IP}}\left(S|c,M,e_{0}\right)=\frac{f_{S}P_{b}\left(S|c,M,E_{0}\right)}{\sum_{\sigma \in D}f_{\sigma }P_{b}\left(\sigma |c,M,E_{0}\right)}\approx \frac{f_{S}\left(e^{E\left(S\right)}+\left(L_{S}-L_{w}+1\right)e^{E_{0}}\right)}{\sum_{\sigma \in D}f_{\sigma }\left(e^{E\left(\sigma \right)}+\left(L_{\sigma }-L_{w}+1\right)e^{E_{0}}\right)},$$ (2.4)

with D being the data set containing all the reads at hand, and $f_{S}$ a frequency prior that corrects for the fact that the pool of oligomers has a non-uniform nucleotide composition. Note that, due to the linearization in c , $P_{\mathrm{IP}}$ is independent of the concentration c since it cancels as an overall prefactor in both numerator and denominator. (2.4) is essentially a formulation of Bayes’ theorem with conditional probability $P_{b}\left(S|c,M,E_{0}\right)$ of having a read S bound by an RBP, the likelihood of finding a read S in the pool washed over the RBP, $f_{S}$ , and an overall normalization (denominator).

Eventually, the logarithmic likelihood of our library of oligomers D reads

$$\log P\left(D\right)\approx \sum_{S\in D}n_{S}\log\left[\frac{f_{S}\left(e^{E\left(S\right)}+\left(L_{S}-L_{w}+1\right)e^{E_{0}}\right)}{\sum_{\sigma \in D}f_{\sigma }\left(e^{E\left(\sigma \right)}+\left(L_{\sigma }-L_{w}+1\right)e^{E_{0}}\right)}\right],$$ (2.5)

where $n_{S}$ is the number of copies of read S in our library.

## 3. Implementation

Our goal is to optimize the parameters in (2.5) such that they maximize the likelihood of our library to be realized in the present way. As a side note, it is equivalent to optimize $P\left(D\right)$ , or its logarithm because the logarithm strictly increases as its argument increases and decreases as its argument decreases. Since the libraries are typically quite big it is beneficial for us to maximize the logarithm in order to keep the overall numbers under control. While the library D , the copy number of a read $n_{S}$ , the read and binding site length $L_{S}$ and $L_{w}$ , and – with some limitations – the frequency priors $f_{S}$ are given from our data, the position-specific binding encoded by the PWM and the position-unspecific binding $e^{E_{0}}$ have to be obtained during the optimization process. Eventually, we want to obtain the the PWM, whereas $e^{E_{0}}$ represents a hidden parameter which will be inferred via the expectation-maximization procedure. In principle, this would also apply to the concentration c but none of our final expressions depend on c any more due to the linearization. Before diving into the details of the EM procedure’s implementation we would like to comment on how to infer the frequency priors $f_{S}$ .

### 3.1. Construction of the frequency priors *f*_*S*_ from a Markov model

RNA Bind’n Seq data does not only comprise libraries of pulled down RBP-bound reads at different, non-vanishing RBP concentrations, but also control experiments that do not contain any RBPs. The oligonucleotides that were used for RBP affinity-based selection were short, typically 20 nucleotides in length (c.f. Ref. 8). The number of possible 20mers is $4^{20}\approx 10^{12}$ , much larger than the library sizes of $∼10^{7}$ . Thus, even in the absence of selection ( c=0 ), the expected overlap of two libraries is extremely small.

To preserve the statistical power of the foreground pool, i.e. use all the reads detected in the foreground sample in the analysis, even though they were not represented in the background sample, we would have to predict the frequency of foreground reads under the assumption of no selection for binding the RBP. A commonly used approach for this type of problem is to train a Markov model from the background pool and construct the expected frequency of each read in the foreground from the trained model, just as in Ref. 9. For an completely unbiased process of oligomer synthesis and capture, the degree d of the Markov model would be 0 , i.e. each base would be equally likely to occur at any position in the oligomer, and all 20mers would have the same prior frequency of occurrence $f_{S}$ . However, biases in the capture and sequencing of oligomers could lead to some sequences, with specific composition of short nucleotide motifs, being captured more often than others. To account for this possibility we trained Markov models of different orders and found that, in general, the higher the order of the model trained from the background sequences, the better the prediction of likelihood of sequences in the foreground samples. Thus, we used a Markov model of order d=14 , which allows the most accurate prediction of background reads frequencies with our computational resources.

### 3.2. Inferring PWMs from the expectation maximization algorithm

Having constructed our model, with the final expression (2.5), and having constructed the background frequencies $f_{S}$ as described in the subsection above, the main remaining question is how to optimize the PWMs and $E_{0}$ such that the likelihood for the result being realized (c.f. (2.5)) is maximized. To this end, we rely on the expectation maximization algorithm. ^10^^,^^11^ Provided that only some of our model parameters can be directly inferred from the data, the algorithm optimizes the “hidden” parameters to maximize (2.5). The expectation-maximization procedure (EM) can be divided into the following steps:

1. Initialize $E_{0}$ and the PWM elements $m_{i}^{\alpha }$ with respectively well-defined real numbers, i.e. $E_{0}\in \left(-\infty ,0\right]$ and $\sum_{\alpha }m_{i}^{\alpha }=1\;\forall \;i=1,\ldots ,L_{w}$ . This can either be done in an entirely unbiased way or by pre-determining some motifs and specifying randomly or uniformly initialized positions in the PWM.
2. Optimize $E_{0}$ to maximize (2.5) holding the PWM fixed.
3. Updating the PWM with the new $E_{0}$ from the previous step. The update of the PWM works by splitting the data set into $L_{w}$ -mers s (on a read S ) and adding the weight

$$\frac{P\left(s|c,M,E_{0}\right)}{P\left(S|c,M,E_{0}\right)}=n_{S}\frac{e^{E\left(s\right)}}{e^{E\left(S\right)}+e^{E_{0}}\left(L_{S}-L_{w}+1\right)}$$ (3.1)

to all entries in the PWM corresponding to s . Repeat that for all s in S , and over all S in D . Renormalize the PWM again by enforcing $\sum_{\alpha }m_{i}^{\alpha }=1\;\forall \;i=1,\ldots ,L_{w}$ .

4. Repeat the previous two steps until convergence. We terminate the iteration when the quadratic difference between the current and the updated PWM is less than $10^{-6}$ on average per entry, i.e. for $L_{w}=5$ the quadratic difference is less than $5\times 4\times 10^{-6}$ . Usually, this takes $O\left(10\right)$ iterations.

Our code is written in C++ and python and is publicly available. ^12^

## 4. Results

**Figure 1.**
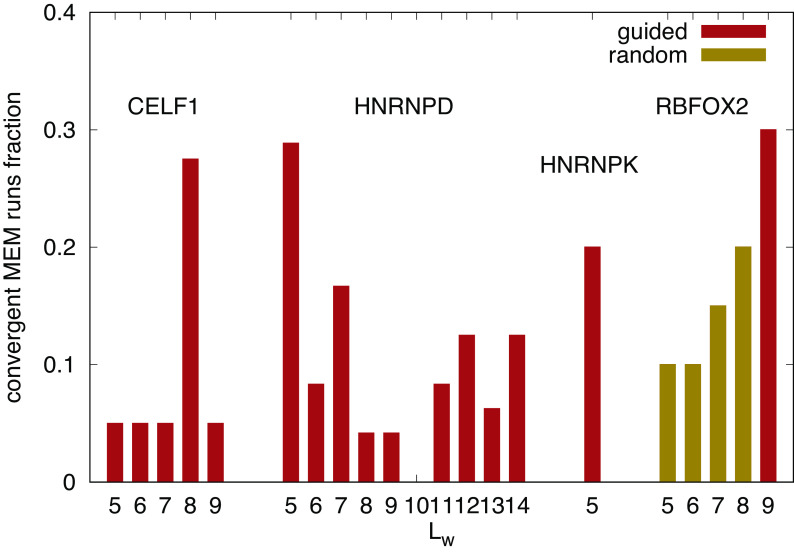
Summary of fraction of convergent outcomes for different investigated RBPs and binding site length $L_{w}$ . RBPs and binding site lengths with no convergent maxima of the log-likelihood (as described in the corresponding subsections, e.g. subsection 4.6) were discarded. While $E_{0}$ is always initialized with a negative random number, the PWM can be initialized either “guided” by already obtained shorter motifs or literature motifs (c.f. corresponding subsection of Sec. 4), or with a fully “random” PWM.

In analyzing Bind’n Seq datasets for various RBPs, we found that only a small subset of random initializations deliver a convergent EM process. The larger $L_{w}$ is the larger is the space of possible initializations, therefore it becomes increasingly unlikely to accidentally hit a region of initialization which converges. This could be compensated for by increasing the number of runs by a factor of 4 for each additional position in the PWM. To avoid that, one can use the knowledge of previous runs, done with shorter PWMs and initialize the longer PWM from the shorter PWM, filling up the additional entries with randomly initialized values to check if the shorter PWM is part of a longer PWM. We carry on with this procedure until the EM algorithm does not find any minimum any more amongst 12 different random initializations of $E_{0}$ and the random initialization of the PWM at the additional positions. Sometimes, neither a fully random intialization nor an initialization of the PWM “guided” by prior knowledge lead the EM algorithm converging to a minimum in log-likelihood (2.5). The relative efficiency of the algorithm finding true maxima is displayed in Figure 1 for the RBPs discussed in the following section. We consider an outcome of the algorithm to be a “true” maximum if the posterior log-likelihood is larger than the initial one and the algorithm is not stuck in a region where $E_{0}$ is large, meaning that the unspecific binding dominates. The maximization algorithm is eventually terminated by a limit of 200 iterations. For readability, we list the used Bind’n Seq data files from Ref. 8 in Table 1 in Sec. 6.

### 4.1 Benchmark: PWM of length 6 for RBFOX2

**Figure 2.**
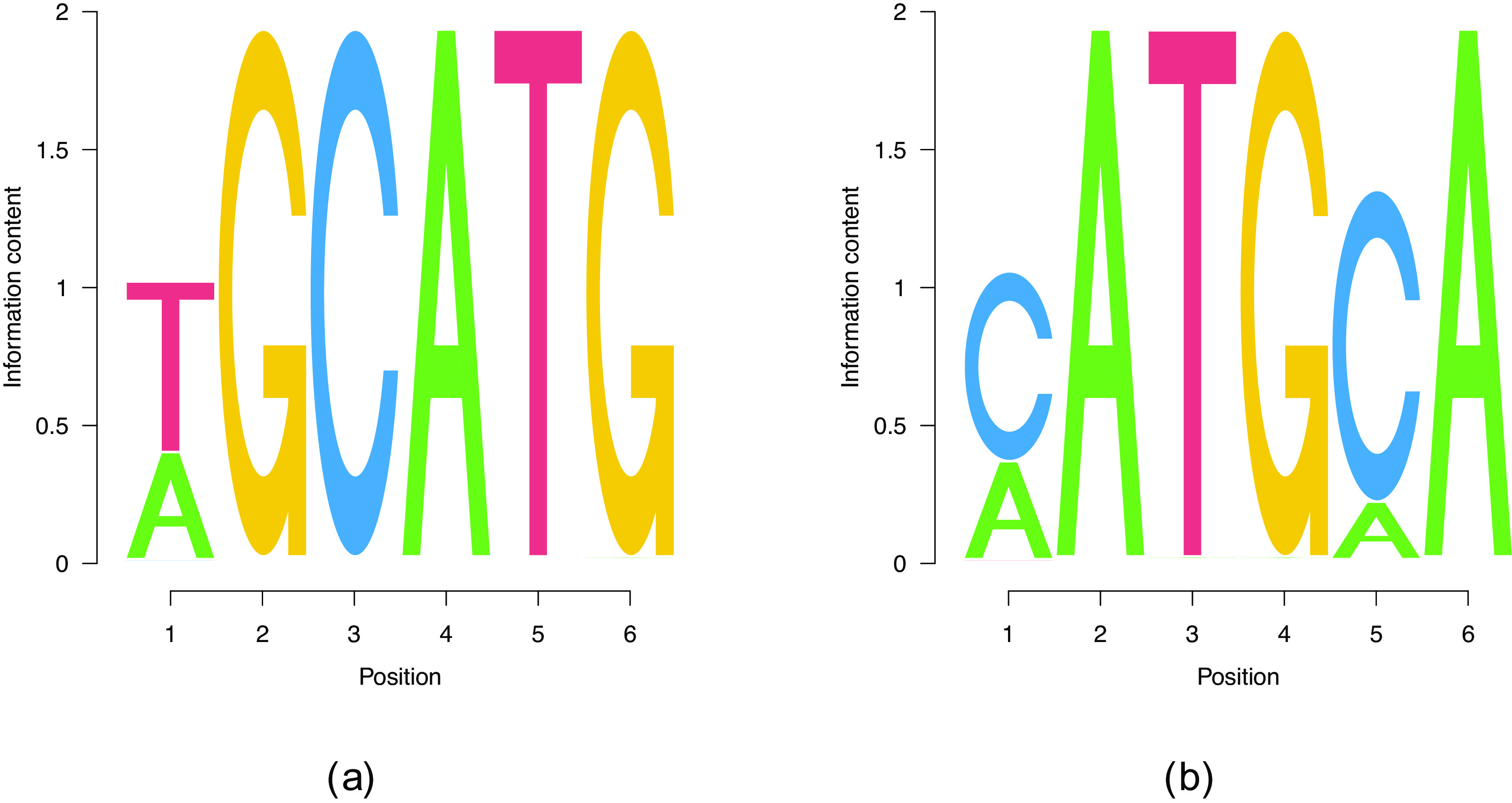
Findings of our model for PWMs of $L_{w}=6$ . (a) The consensus motif that features a higher $\log P\left(D\right)$ than (b). However, (b) is also present.

To benchmark our method, we started our evaluation with RBFOX2, a key regulator of alternative splicing ^13^ that was extensively studied with a variety of methods (e.g. Ref. 14). The RBFOX2 Bind’n Seq dataset ^8^ consists in nine libraries at nine different protein concentrations and two protein-free control libraries, all containing reads of 50 nucleotides (nts) in length, including the adaptor. RBFOX2 is widely used to benchmark computational analysis methods (c.f. Ref. 15) and thus the corresponding dataset was carefully generated, to include multiple, high-quality libraries. Established techniques like kmer-enrichment analysis and the streaming-kmer-algorithm (SKA) predict a consensus 6mer TGCATG as the most prominent motif followed by other GCATG-containing 6mers. ^15^ Our results in Figure 2 reproduce the predicted TGCATG 6mer as a part of the motif Figure 2(a). Moreover, we find the subdominant PWM Figure 2(b) which has a quite substantial overlap with Figure 2(a) in the first four positions. We therefore consider an important real world data test of our code to be passed.

### 4.2 Other PWMs found for RBFOX2

**Figure 3.**
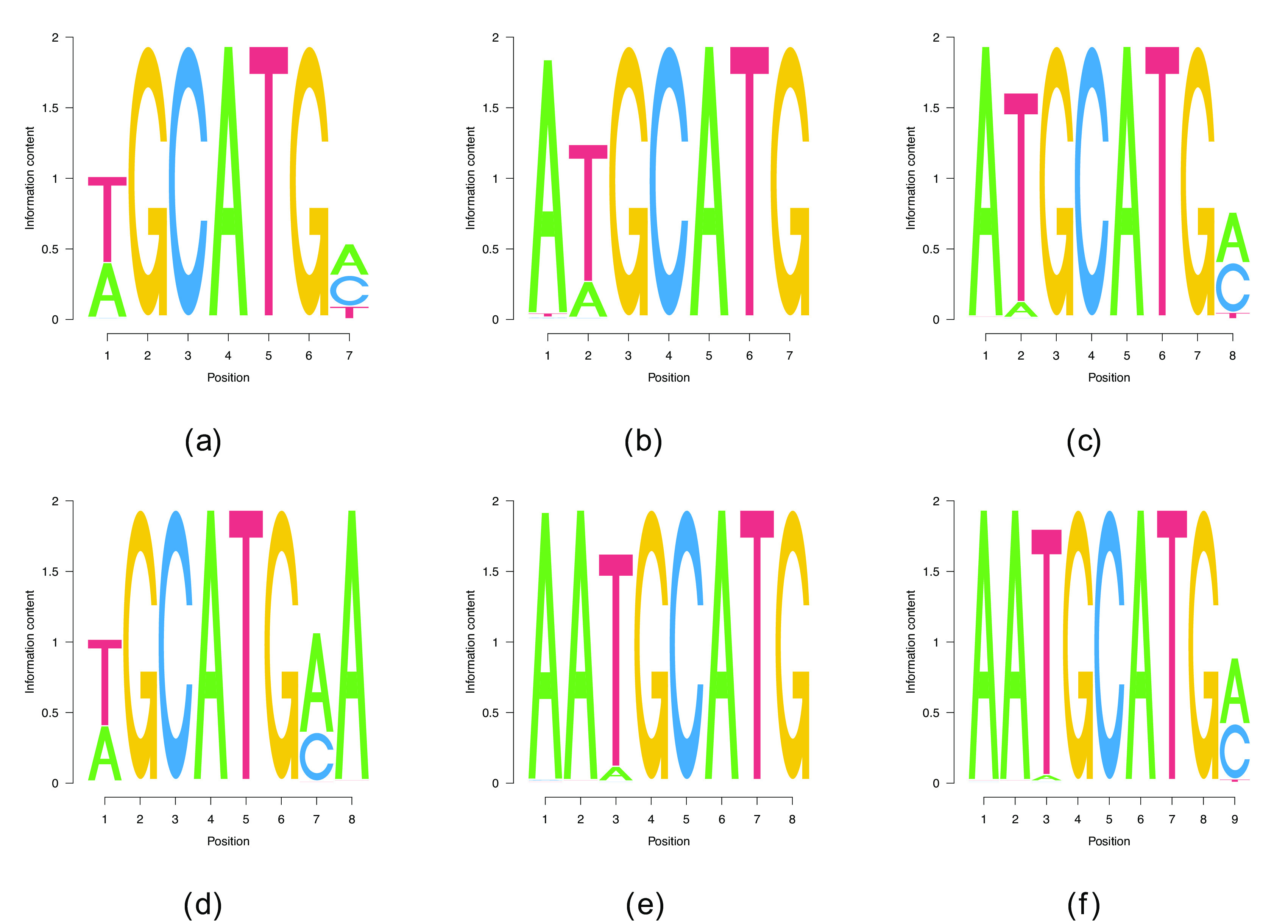
Non-consensus PWMs of different $L_{w}$ for RBFOX2.

The big overlap of the motifs Figure 2 suggests also searching for longer motifs, which may subsume the shorter ones. Indeed, the motif shown in Figure 3(d) contains both 6mers. Along with that we find local minima in the probability landscape, i.e. PWMs of $L_{w}\leq 9$ (see Figure 3). All motifs have the consensus TGCATG in common. For RBFOX2, we found no evidence for our model to converge beyond $L_{w}=9$ . The posterior probability $\log\hat{P}\left(D\right)$ – (2.5) at the optimized parameters – serves as a measure to compare and rank different motifs at equal $L_{w}$ . The Bayesian Information Criterion (BIC) ^16^ estimates the information content of every obtained local minimum,

$$\mathrm{BIC}=k\log n-2\log\hat{P}\left(D\right),$$ (4.1)

with the number of degrees of freedom $k=\left(4-1\right)L_{w}+1$ (four nucleotides minus one for the normalization per position, one extra for $E_{0}$ ), and the number of data points $n=\sum_{S}n_{S}\left(L_{S}-L_{w}+1\right)$ , i.e. the number of possible binding sites in the entire foreground pool. Closely related is the Akaike Information Criterion (AIC) ^17^

$$\mathrm{AIC}=2k-2\log\hat{P}\left(D\right),$$ (4.2)

which is a bit less susceptible to overfitting. Both criteria rank the longer PWMs as having the higher information content, while we rather expect to find an optimum in information content with respect to $L_{w}$ . Therefore, we compare posterior probabilities only among equal $L_{w}$ in the following and leave the search for a comparison criterion among different $L_{w}$ for future work. Having found that the model retrieves the expected motif for a well-studied RBP, we sought to further explore its performance for others.

### 4.3 CELF1

**Figure 4.**
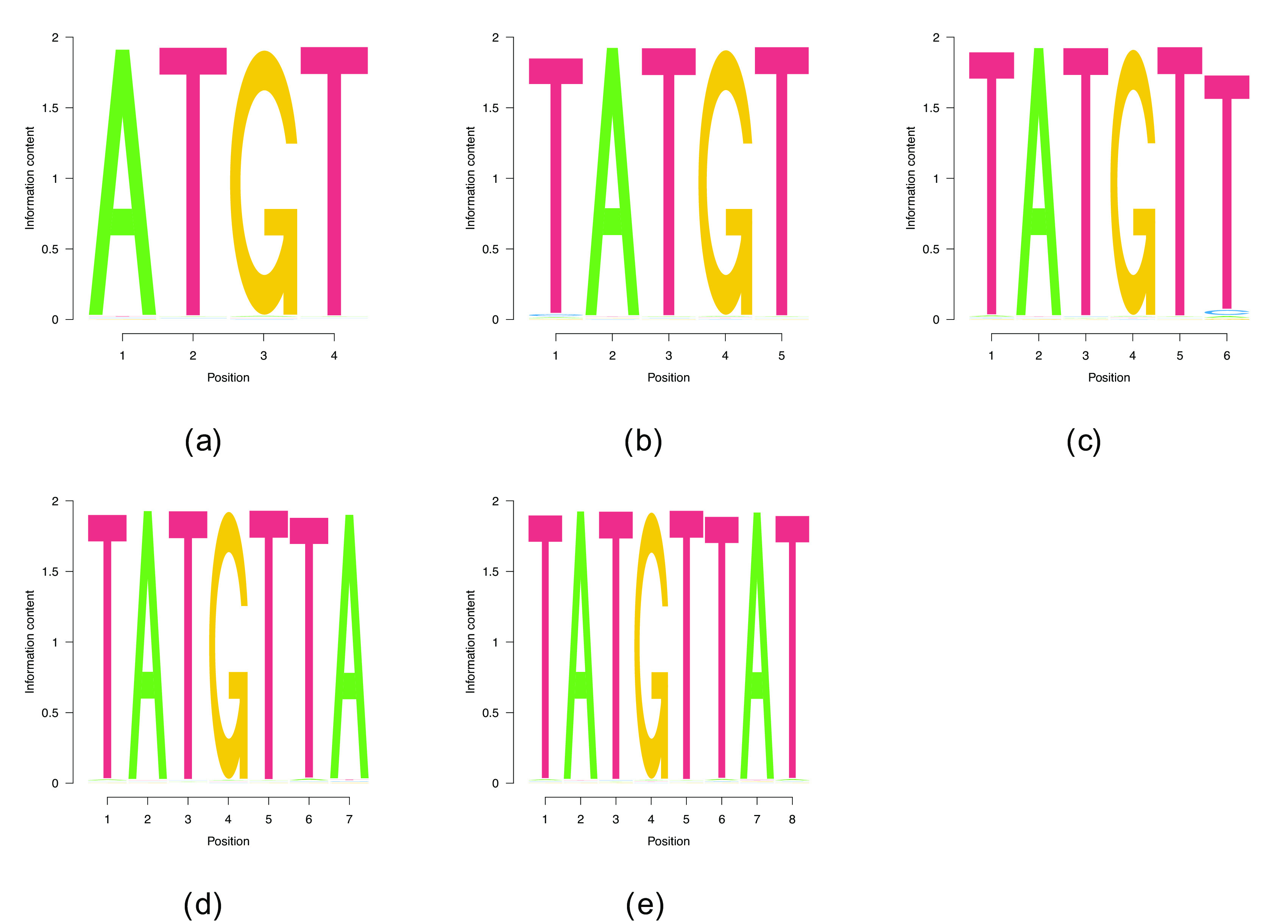
PWMs of different $L_{w}$ for CELF1.

CELF1 is an RBP of the CUG-binding CELF family. ^18^ CELF1 participates in multiple steps of post-transcriptional processing of RNAs, including splicing, translation and decay, ^19^ and requires UGU motifs for high-affinity interaction with RNAs. ^20^ The corresponding Bind’n Seq dataset ^8^ consists of libraries generated for seven different RBP concentrations, each containing $∼2\times 10^{7}$ reads of $L_{S}=40$ . Since 40 runs with completely random PWM intialization for $L_{w}=3,4,5$ did not yield any local optima of the probability landscape we decided to test whether the biased initialization of the PWM with the known motif ( UGU / TGT ), which was found as as enriched 3-mer in RNA Bind’n Seq ^8^ enables the recovery of longer motifs. Indeed, our procedure yielded multiple extended versions of TGT Figure 4 with $L_{w}$ up to 8 . The context in which the reduced motif occurs is A/T-rich, the presence of an A immediately upstream indicates that CELF1 could recognize the AUG start codon. This could be interesting in light of CELF1 being a translational regulator of epithelial-mesenchymal transition via the binding of both cap-binding EIF4E and the poly(A)-binding protein. ^21^

### 4.4 HNRNPD

**Figure 5.**
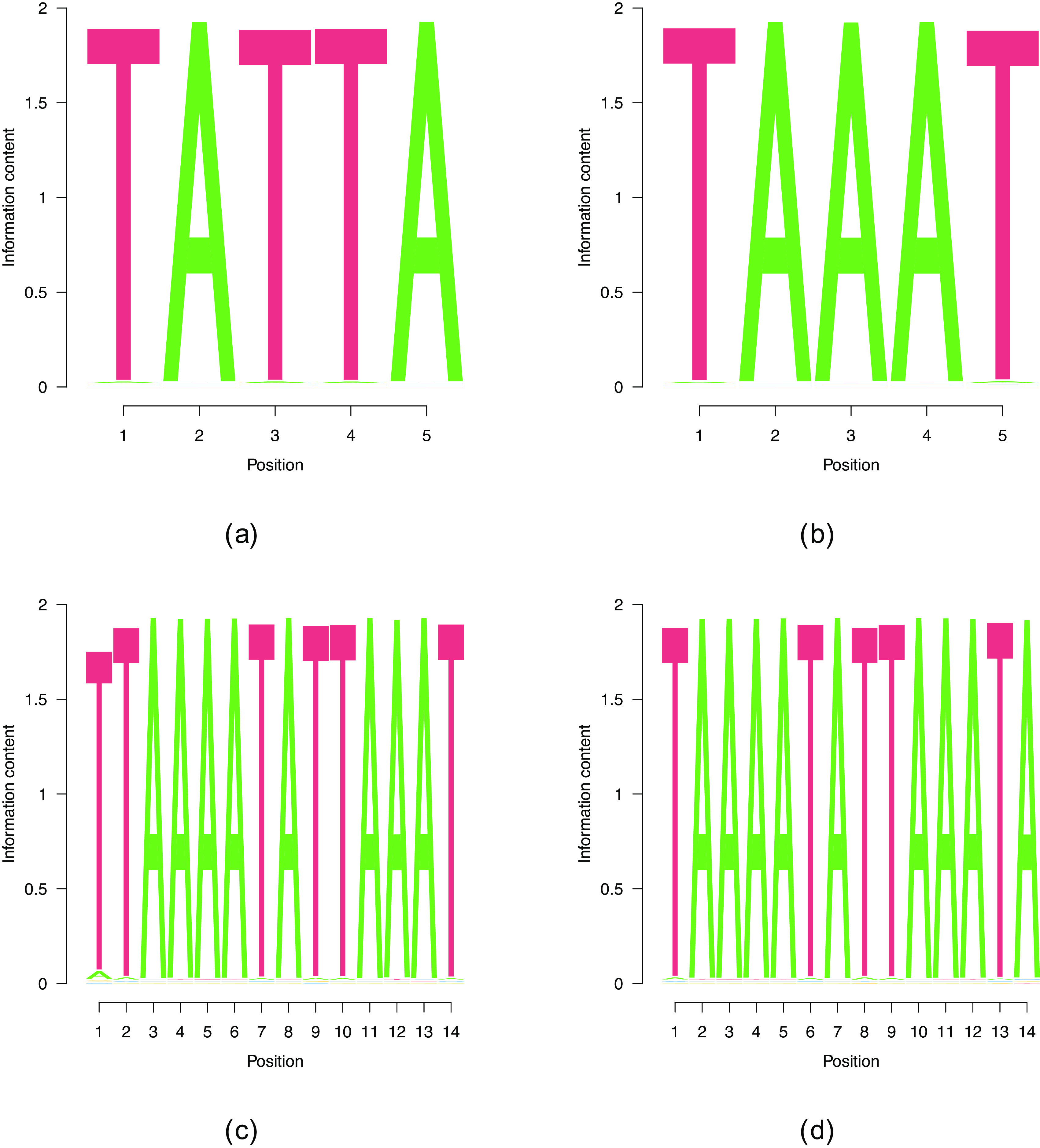
PWMs of different $L_{w}$ for HNRNPD. The benchmark cases $L_{w}=5$ are show in (a) and (b), whereas (c) and (d) show the two motifs of $L_{w}=14$ , the longest motifs that our algorithm found.

Within the class of heterogeneous ribonucleoproteins (hnRNPs), hnRNPD (also known as AUF1) is a well-known A/U-rich element RNA binding protein with important role in RNA decay. ^22^ HNRNPD has been reported to bind clusters of AUUUA elements. ^22^ The ENCODE-database ^8^ lists AUAAU as another possible binding site for hnRNPD. While entirely random initializations do not deliver any convergent runs, we recover both AUAAU, and, with a smaller binding log-likelihood, AUUUA, as binding sites. Building on this shorter motifs enables the discovery of UAAAU-containg longer motifs that can be extended up to $L_{w}=14$ (see Figure 5), the highest length for which we found convergent results. We did find PWMs with $L_{w}=7,\ldots ,13$ which we omitted in Figure 5 since they are parts of the two $L_{w}=14$ PWMs.

### 4.5 HNRNPK

We were also interested in determining whether we can recover G/C-rich binding motifs from the data and therefore applied the model to heterogeneous nuclear ribonucleoprotein K (HNRNPK), a member of the poly(C) binding family of proteins. ^23^ We could only recover one of the two consensus motifs reported in the ENCODE analysis of these data (GCCCA, from SKA ^8^) when initialized with it. The second reported motif, with the CACGC consensus, could not be found by our algorithm even when the PWM was initialized with the motif itself and even when sequences containing the first motif were eliminated, indicating that this motif does not correspond to a local maximum of the likelihood function. We did not find any PWMs of $L_{w}>5$ in this data set, whether we used random initialization or shorter motif-guided initialization.

### 4.6 Other RBPs

**Figure 6.**
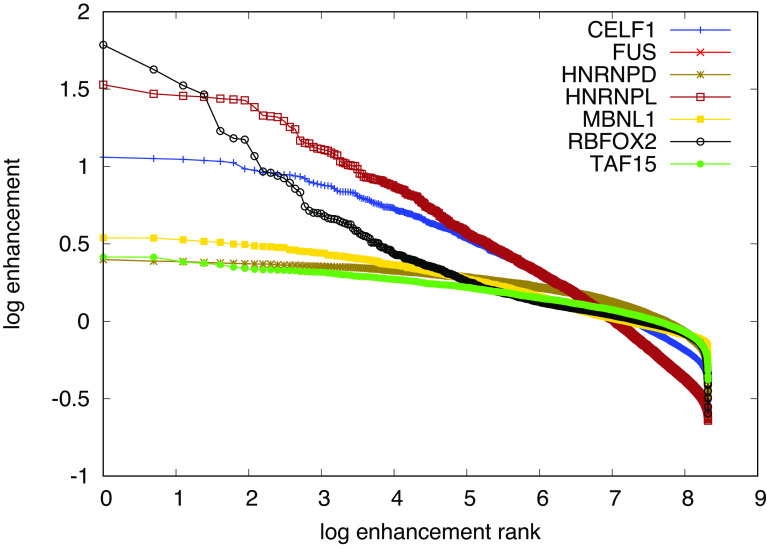
Logarithmic (base eenhancement (counts foreground normalized by counts background) of all $4^{6}\approx 4000$ possible 6mers of all investigated RBPs, ranked by enhancement. The top most enhanced motifs are RBFOX2: TGCATG, FUS: GCGCGC, hnRNPL: CACACA, MBNL1: GCTGCT, TAF15: GGGGGG, followed by variants thereof. All except the RBFOX2 motif are repeats of shorter oligomers.

There are other proteins covered in the Bind’n Seq data ^8^ whose specificity was studied before. For example, we analyzed the data corresponding to MBNL1, ^24^ hnRNPL, ^25^ FUS, ^26^ TAF15. ^27^ For these, our model did not deliver any convergent results, even if the PWM was directly intialized with the expected consensus motif. This indicates that the enrichment did not work equally well for all the RBPs studied with the Bind’n Seq method. Interestingly, expected motifs were identified for these proteins with another method, the so-called kmer-enhancement that underlies most of the consensus motifs reported in the ENCODE database. ^8^ Kmer enhancements are computed by counting the number of occurrences of every possible kmer in the foreground samples (RBP concentration ≠0 ) and in the background samples (RBP concentration =0 ), and finally normalizing the foreground abundances by the background to extract the respective enhancement. The higher the enhancement of a given kmer, the higher the likelihood of it being bound by the RBP used in the experiment is thought to be. We computed these enhancements for 6mers, as done in the ENCODE studies. The results, shown in Figure 6 indicate that only RBFOX2 has a few highly enhanced 6mers with a clear hierarchy of enrichment, while all other investigated RBPs show a much flatter hierarchy of motif enhancements. An analysis of the Levenshtein distance of these motifs showed no clear difference in the pattern of distances among the leading motifs across the investigated RBPs. This suggests that these motifs correspond to many local minima of comparable depth, which precludes our algorithm finding clear PWMs representing the binding sites. Conversely, it becomes unclear whether the specificity of these RBPs would be well represented as weight matrices, or whether another model, for e.g. clusters of short, degenerate motifs may better represent the specificity of these RBPs.

## 5. Conclusion

We constructed a thermodynamical model that can be used to infer characteristic position weight matrices for the binding domains of RNA binding proteins from data obtained from affinity-based enrichment of oligonucleotides. Since we directly model the RBP-binding specificity as PWMs, our method bypasses arbitrary choices in the alignment of k-mers found to be individually enriched in the data. We evaluate our model on data in the public domain ^8^ using expectation-maximization. For the benchmark case of RBFOX2, where very high-quality data is available, our model reproduces the known binding motif TGCATG where the first position features an almost uniform superposition of T and A in our result. Subdominantly, we find another PWM of $L_{w}=6$ as well as longer PWMs for RBFOX2. Unfortunately, our principled model does not robustly recover the binding motifs of other RBPs. For a few, e.g. CELF1, HNRNPD, HNRNPK, we can still recover the motifs as well as some longer variants, if the search starts from a PWM closely matching the expected motif. However, for most of the data sets our model did not deliver any prediction. Rather, other motifs, e.g. poly(A), often show higher enrichment in the data than the expected motifs. This indicates that experimental details that do not have to do with the affinity of the RBP for oligomers affect the frequency of oligomer capture in the data, complicating its analysis and raising questions about the biophysical realism of the motifs derived from the data. The motifs of the RBPs for which we did not recover a PWM tend to be more degenerate than those of RBPs for which some motif emerged. They consist of repeated occurrences of mono, di or trinucleotides. It is likely that for these motifs, it is crucially important to construct an appropriate background model. How to best do this remains to be determined in future work. Of note, while crosslinking and immunoprecipitation data is available for the proteins studied here, PWMs with enriched binding sites were also not recovered. ^28^ Thus, it will be interesting to explore models that allow more flexible spacing of RBP contact points on RNAs in the future.

## Software availability

Source code available from: https://git.scicore.unibas.ch/zavolan_group/pipelines/bind-n-seq-pwms

Archived source code at time of publication: https://doi.org/10.5281/zenodo.8028034 ^29^

License: MIT
