# On the limits of inferring biophysical parameters of RBP-RNA interactions from in vitro RNA Bind’n Seq data

**DOI:** 10.12688/f1000research.135164.2

**Published:** 2024-05-29

**Authors:** Niels Schlusser, Mihaela Zavolan

**Affiliations:** 1 Biozentrum, Universitat Basel, Basel, Basel-Stadt, 4056, Switzerland

**Keywords:** Systems biology, bioinformatics, computational biology, machine learning, maximum entropy method, Bayesian statistics, RNA binding proteins, RNA Bind'n'Seq

## Abstract

We develop a thermodynamic model describing the binding of RNA binding proteins (RBP) to oligomers *in vitro.* We apply expectation-maximization to infer the specificity of RBPs, represented as position-specific weight matrices (PWMs), by maximizing the likelihood of RNA Bind’n Seq data from the ENCODE project. Analyzing these public data we find sequence motifs that can partly explain the data for more than half of the studied 111 RBPs, and for 48 of the proteins these motifs are consistent with the known specificity. Our code is publicly available, facilitating analysis of RBP binding data.

## 1. Introduction

RNA-binding proteins (RBPs) interact with RNAs at every step of their life cycle. Due to their modular structure, usually consisting in an assortment of RNA-binding domains, RBPs interact with both RNAs and proteins, and couple various layers of gene expression. ^1^ While ∼2500 RBPs are currently known, most remain to be functionally characterized. A first step in this process is to determine the interaction partners and the sequence/structure specificity of the RBP. Many RBPs recognize their targets in a sequence-specific manner, although the accessibility of binding sites within the targets also plays a role. ^2^ The sequence specificity is usually represented by a position weight matrix (PWM), which specifies the probability of finding each of the four nucleotides at each position in the RBP binding site. This is an obvious simplification, as dependencies between positions in the binding site likely occur. However, training more complex models requires substantially more data, which are often not available. Moreover, in the case of another class of nucleic acid binding proteins, transcription factors, the improvement in binding site predictability by more complex models is modest, at least in the case of other nucleic acid binding proteins, transcription factors. ^3^ With the realization that the presence of a canonical RNA-binding domains is not necessary for the ability of a protein to bind RNAs ^4^ came a pressing need to determine the determinants of RNA-RBPs interactions and the sequence/structure specificity of the proteins newly found to interact with RNAs.

The past two decades have seen the development and broad application of experimental methods for RBP target identification. They include *in vivo* high-throughput approaches such as HITS-CLIP, PAR-CLIP, iCLIP and eCLIP (reviewed in Refs. 5, 6), and more recently-developed *in vitro* approaches such as RNA Bind’n Seq. ^7^ While the CLIP methods rely on the sequencing of RNAs that interact with and can therefore be crosslinked to RBPs *in vivo*, RNA Bind’n Seq relies on the affinity-dependent interaction of RBPs with random RNAs *in vitro.* The oligonucleotides selected in this experiment are computationally analyzed to identify short sequence motifs that mediate the interaction with the RBP. So far, analyses of such data involved the identification of enriched kmers (short oligonucleotide sequences of a specified length, k ), and then a greedy alignment procedure yielded PWM representations of the RBP binding motifs. This left open the question of whether the derived PWMs accurately predicted the interaction energies of RBPs with their binding sites. In contrast, the aim of our work was to develop a biophysics-anchored method to directly infer the PWMs from RNA Bind’n Seq data. Our paper is organized as follows: Sec. 2 explains how we derive our thermodynamical model. We comment on the practical implementation of this model in Sec. 3, where we also explain how we account for sequence composition biases in the pool of oligomers. Results for different RBPs are presented in Sec. 4, where we also comment on the accuracy of the results obtained from this type of data for different RBPs. Concluding remarks are given in Sec. 5.

## 2. Model

Our model is an adaptation of a Bayesian, thermodynamic model that was constructed to infer di-nucleotide weight tensors from SELEX data. ^8^ In the following, we derive the log-likelihood of Bind’N Seq data given the PWM for the RBP of interest, which will be inferred by expectation-maximization as described in Sec. 3.

### 2.1 Derivation of the data likelihood

We assume that an RBP binds an oligomer over a binding site s of length $L_{w}$ and that the likelihood of binding taking place, according to Boltzmann’s law, goes as $\propto \exp\left(\sum_{i=1}^{L_{w}}E_{i}^{s_{i}}\right)\equiv e^{E\left(s\right)}$ , where $s_{i}$ is the nucleotide at position i , so $s_{i}\in \left\{A,C,G,T\right\}$ . Therefore, each element of the position weight matrix (PWM) M can be identified with $m_{i}^{\alpha }\equiv \exp\left(E_{i}^{\alpha }\right)$ , with columns being normalized as $\sum_{\alpha }m_{i}^{\alpha }=1\;\forall \;i=1,\ldots ,L_{w}$ .

Additionally, we account for the fact that there are genuinely two different ways of binding, sequence-specific binding as described by the PWM, and unspecific binding to RNAs with a probability $\exp\left(E_{0}\right)$ . Combining these two possibilities, we arrive at the probability of a site s being bound by the RBP

$$P\left(\text{bound}|s,c,M,E_{0}\right)=\frac{c\left(e^{E\left(s\right)}+e^{E_{0}}\right)}{1+c\left(e^{E\left(s\right)}+e^{E_{0}}\right)},$$ (2.1)

where the 1 in the denominator represents the (constant) chance of the RBP being unbound. Assuming that the binding of RBPs to oligomers is not saturated, i.e. $c\left(e^{E\left(s\right)}+e^{E_{0}}0\right)\ll 1$ , we can linearize (2.1)

$$P\left(\text{bound}|s,c,M,E_{0}\right)\approx c\left(e^{E\left(s\right)}+e^{E_{0}}\right).$$ (2.2)

Consequently, the chance of an RBP being bound somewhere on a longer oligomer S with $L_{S}\geq L_{w}$ is

$$P\left(\text{bound}|S,c,M,E_{0}\right)=\sum_{s\in S}P\left(\text{bound}|s,c,M,E_{0}\right)\approx c\left(e^{E\left(S\right)}+\left(L_{S}-L_{w}+1\right)\;e^{E_{0}}\right),$$ (2.3)

where $e^{E\left(S\right)}\equiv \sum_{s\in S}e^{E\left(s\right)}$ is the sum over all possible $L_{w}$ -mers s in S . The probability to observe each read S in the pool of oligomers that were selected by the interaction with the RBP is

$$\begin{array}{c} P\left(\mathrm{IP}|S,c,M,E_{0}\right)=\frac{f_{S}P\left(\text{bound}|S,c,M,E_{0}\right)}{\sum_{\sigma \in D}f_{\sigma }P\left(\text{bound}|\sigma ,c,M,E_{0}\right)}\\ \;\approx \frac{f_{S}\left(e^{E\left(S\right)}+\left(L_{S}-L_{w}+1\right)\;e^{E_{0}}\right)}{\sum_{\sigma \in D}f_{\sigma }\left(e^{E\left(\sigma \right)}+\left(L_{\sigma }-L_{w}+1\right)\;e^{E_{0}}\right)}, \end{array}$$ (2.4)

with D being the data set of reads, IP being a binary variable indicating if the read is immuno-percipitated or not, and $f_{S}=P\left(S\right)$ a frequency prior that corrects for the fact that the pool of oligomers has a non-uniform nucleotide composition. Note that, due to the linearization in c , $P\left(\mathrm{IP}|S,M,E_{0}\right)$ is independent of the protein concentration c , which cancels out as an overall prefactor in both numerator and denominator. (2.4) is essentially a formulation of Bayes’ theorem, with $P\left(\mathrm{IP}|S,M,E_{0}\right)$ being the likelihood of having a read S being immuno-percipitated, $P\left(S\right)=f_{S}$ being the likelihood of the read S in the input, and having an overall normalization in the denominator.

Eventually, the log-likelihood of the entire library of oligomers D explained by the specific binding to the RBP described by the PWM M of length $L_{w}$ as well as unspecific binding described by parameter $E_{0}$ is given by

$$\log P\left(D|M,E_{0}\right)\approx \sum_{S\in D}n_{S}\log\left[\frac{f_{S}\left(e^{E\left(S\right)}+\left(L_{S}-L_{w}+1\right)\;e^{E_{0}}\right)}{\sum_{\sigma \in D}f_{\sigma }\left(e^{E\left(\sigma \right)}+\left(L_{\sigma }-L_{w}+1\right)\;e^{E_{0}}\right)}\right],$$ (2.5)

where $n_{S}$ is the number of copies of read S in the library.

### 2.2 Relationship to the dissociation constant *K*_*D*_

The log-likelihood of binding derived in the previous section can be related to the dissociation constant $K_{D}$ known from the formalism of chemical reactions as follows. The concentration ${\left[\mathrm{RBP}\right]}_{\text{bound}}$ of RBP bound to a multivalent oligomer with concentration [S] is given by ^9^

$${\left[\mathrm{RBP}\right]}_{\text{bound}}=\frac{n\left[S\right]}{\frac{K_{D}}{\left[\mathrm{RBP}\right]}+1},$$ (2.6)

where $K_{D}$ is the dissociation constant and n is the number of binding sites on the oligomer. We make the simplification of considering binding sites to be in principle identical and their maximum number be given by the total number of configurations in which the RBP can contact the oligomer, i.e. $L_{S}-L_{w}+1$ .

Further assuming that the probability to observe individual oligomers in the sequencing pool is proportional to their relative abundance in complex with the RBP (which can only hold when at most one RBP molecule is bound to an individual oligomer), i.e. $P\left(S|M,E_{0}\right)\propto {\left[\mathrm{RBP}\right]}_{\text{bound}}$ , we can re-express (2.5) in terms of (2.6) as

$$\begin{array}{l} \log P\left(D|M,E_{0}\right)=C+\sum_{S\in D}n_{S}\log\left(\frac{n\left[S\right]}{\frac{K_{D}}{\left[\mathrm{RBP}\right]}+1}\right)\\ \;=C+\sum_{S\in D}n_{S}\log\left(\frac{f_{S}\left(L_{S}-L_{w}+1\right)}{\frac{K_{D}}{\left[\mathrm{RBP}\right]}+1}\right), \end{array}$$ (2.7)

where we used that the concentration [S] of any given oligomer S is proportional to $f_{S}$ , C is a proportionality constant independent of the binding specificity. Under the assumption that the binding is typically of low affinity, i.e. $\frac{1}{\frac{K_{D}}{\left[\mathrm{RBP}\right]}+1}\approx \frac{\left[\mathrm{RBP}\right]}{K_{D}}$ , we arrive at

$$\log P\left(D|M,E_{0}\right)\approx C^{\prime }+\sum_{S\in D}n_{S}\log\left(\frac{\left[\mathrm{RBP}\right]}{K_{D}}f_{S}\left(L_{S}-L_{w}+1\right)\right).$$ (2.8)

Hence, we can rank interactions by the dissociation constants relative to some reference PWM $M^{\mathrm{ref}}$ and $E_{0}^{\mathrm{ref}}$

$$\log\left(\frac{K_{D}}{K_{D}^{\mathrm{ref}}}\right)=\frac{1}{\sum_{S\in D}n_{S}}\left[\log P\left(D|M^{\mathrm{ref}},E_{0}^{\mathrm{ref}}\right)-\log P(D|M,E_{0})+\sum_{S\in D}n_{S}\log\left(\frac{L_{S}-L_{w}+1}{L_{S}-L_{w}^{\mathrm{ref}}+1}\right)\right].$$ (2.9)

As expected, the result corrects for the library size-dependence of $P\left(D|M,E_{0}\right)$ by dividing by the total foreground reads $\sum_{S\in D}n_{S}$ , and for differences in binding domain or read length by the last term. Assuming a random oligomer of length $L_{w}^{\mathrm{ref}}$ for which there is no unspecific binding ( $E_{0}^{\mathrm{ref}}\to -\infty$ ) as reference allows us to bring $\log P\left(D|M^{\mathrm{rel}}\right)$ into the simple closed form

$$\log P\left(D|M^{\mathrm{ref}},E_{0}\right)=\sum_{S\in D}n_{S}\log\left[\frac{f_{S}\left(L_{S}-L_{w}^{\mathrm{ref}}+1\right)}{\sum_{\sigma \in D}f_{\sigma }\left(L_{\sigma }-L_{w}^{\mathrm{ref}}+1\right)}\right].$$ (2.10)

Combining (2.9) with (2.5), the output of the optimization procedure, and the reference (2.10) allows us to compute the logarithm of the dissociation constant of the RBP-RNA binding for a representative RBP binding site relative to a random oligomer of length $L_{w}^{\mathrm{ref}}=5$ , a typical size for an RBP binding site. Relative $K_{D}$ ’s also provide a measure to rank different binding specificities, even of different lengths, for a given RBP.

## 3. Implementation

Our goal is to identify the parameters that maximize the likelihood of the library of oligomers given in (2.5). This is equivalent to optimizing P(D) , or rather its logarithm to avoid underflows. While the library D , the copy number of a read $n_{S}$ , the read and binding site lengths $L_{S}$ and $L_{w}$ , and – with some limitations – the frequency priors $f_{S}$ are given from our data, the position-specific binding of the RBP described by the PWM and the position-unspecific binding described by $E_{0}$ have to be inferred. Eventually, we want to obtain the PWM, whereas $E_{0}$ represents a hidden parameter which will be inferred via the expectation-maximization procedure. In principle, this would also apply to the concentration c but none of our final expressions depend on c any more due to linearization. Before diving into the details of the EM procedure’s implementation we would like to comment on how to infer the frequency priors $f_{S}$ .

### 3.1 Construction of the frequency priors *f*_*S*_ from a Markov model

RNA Bind’n Seq data does not only comprise libraries of reads pulled down by specific RBPs at non-vanishing RBP concentrations, but also libraries of input reads. The oligonucleotides that were used for RBP affinity-based selection were short, typically 20 nucleotides in length (c.f. Ref. 10). The number of possible 20mers is $4^{20}\approx 10^{12}$ , much larger than the library sizes of $∼10^{7}$ . Thus, even in the absence of selection ( c=0 ), the expected overlap of two libraries is extremely small.

To preserve the statistical power of the foreground pool, i.e. use all the reads detected in the foreground sample in the analysis, even though they were not represented in the background sample, we have to predict the frequency of foreground reads under the assumption of no selection for binding the RBP. A commonly used approach for this type of problem is to train a Markov model from the background pool and construct the expected frequency of each read in the foreground from the trained model, just as in Ref. 11. For an completely unbiased process of oligomer synthesis, capture and sequencing the degree d of the Markov model would be 0 , i.e. each base would be equally likely to occur at any position in the oligomer, and all 20mers would have the same prior frequency of occurrence $f_{S}$ . However, various types of biases can lead to some sequences, with specific composition of short nucleotide motifs, being present in the data more often than others. In principle, the higher the degree of the Markov model, the larger the sequence context that can be resolved. However, an over-parametrization of the Markov-model needs to be avoided. As binding specificities of RBPs tend to be short and our main aim is to appropriately detect enrichment of motifs on this length scale, we used a Markov model of d=4 , which seemed to give a good tradeoff between the accuracy of the background read frequency prediction, size of Bind’n Seq libraries, and available compuational resources. The predicted frequency priors $f_{S}$ – and kmer-frequencies, accordingly – need to be normalized such that their sum over the background frequency pool B satisfies $\sum_{S\in B}f_{S}=1$ .

### 3.2 Inferring PWMs by expectation maximization

Having constructed our model with the final expression (2.5), as well as the background frequencies $f_{S}$ described in the subsection above, the remaining task is to identify the PWM and $E_{0}$ maximize the log-likelihood (2.5). To this end, we rely on the expectation maximization algorithm. ^12^^,^^13^ Provided that only some of our model parameters can be directly inferred from the data, the algorithm optimizes the”hidden” parameters to maximize (2.5). The expectation-maximization procedure (EM) can be divided into the following steps:

1. Initialize $E_{0}$ and the PWM elements $m_{i}^{\alpha }$ with respectively well-defined real numbers, i.e. $E_{0}\in \left(-\infty ,0\right]$ and $\sum_{\alpha }m_{i}^{\alpha }=1\;\forall \;i=1,\ldots ,L_{w}$ . This can either be done in an entirely unbiased way or by pre-determining some motifs and initializing PWMs with values reflecting those motifs.
2. Recalculate $E_{0}$ to maximize (2.5) holding the PWM fixed, which amounts to finding the root of
$$\begin{array}{l} \frac{\partial }{\partial E_{0}}\log P\left(D|M,E_{0}\right)=\sum_{S\in D}n_{S}\;\frac{\left(L_{S}-L_{w}+1\right)e^{E_{0}}}{e^{E\left(S\right)}+\left(L_{S}-L_{w}+1\right)e^{E_{0}}}\\ \;-\sum_{S\in D}n_{S}\;\frac{\sum_{\rho \in D}f_{\rho }\left(L_{\rho }-L_{w}+1\right)e^{E_{0}}}{\sum_{\sigma \in D}f_{\sigma }\left(e^{E\left(\sigma \right)}+\left(L_{\sigma }+L_{w}+1\right)e^{E_{0}}\right)}\\ \;\overset{!}{=}\;0. \end{array}$$ (3.1)
  We employ a Brent minimization algorithm from the GSL library ^14^ to the negative value of the log-likelihood in (2.10) to maximize it.
3. Updating the PWM with the new $E_{0}$ from the previous step, splitting the data set into $L_{w}$ -mers s (on a read S ) and adding the weight
$$\frac{P\left(s|c,M,E_{0}\right)}{P\left(S|c,M,E_{0}\right)}=n_{S}\frac{e^{E\left(s\right)}}{e^{E\left(S\right)}+e^{E_{0}}0\left(L_{S}-L_{w}+1\right)}$$ (3.2)
  to all entries in the PWM corresponding to s . Repeat that for all s in S , and over all S in D . Renormalize the PWM again by enforcing $\sum_{\alpha }m_{i}^{\alpha }=1\;\forall \;i=1,\ldots ,L_{w}$ .
4. Repeat the previous two steps until convergence. We terminate the iteration when the quadratic difference between the current and the updated PWM is less than $10^{-6}$ on average per entry, i.e. for $L_{w}=5$ the quadratic difference is less than $5\times 4\times 10^{-6}$ . Usually, this takes O(10) iterations.

Our code is written in C++ and python and is publicly available. ^15^

## 4. Results

**Figure 1.**
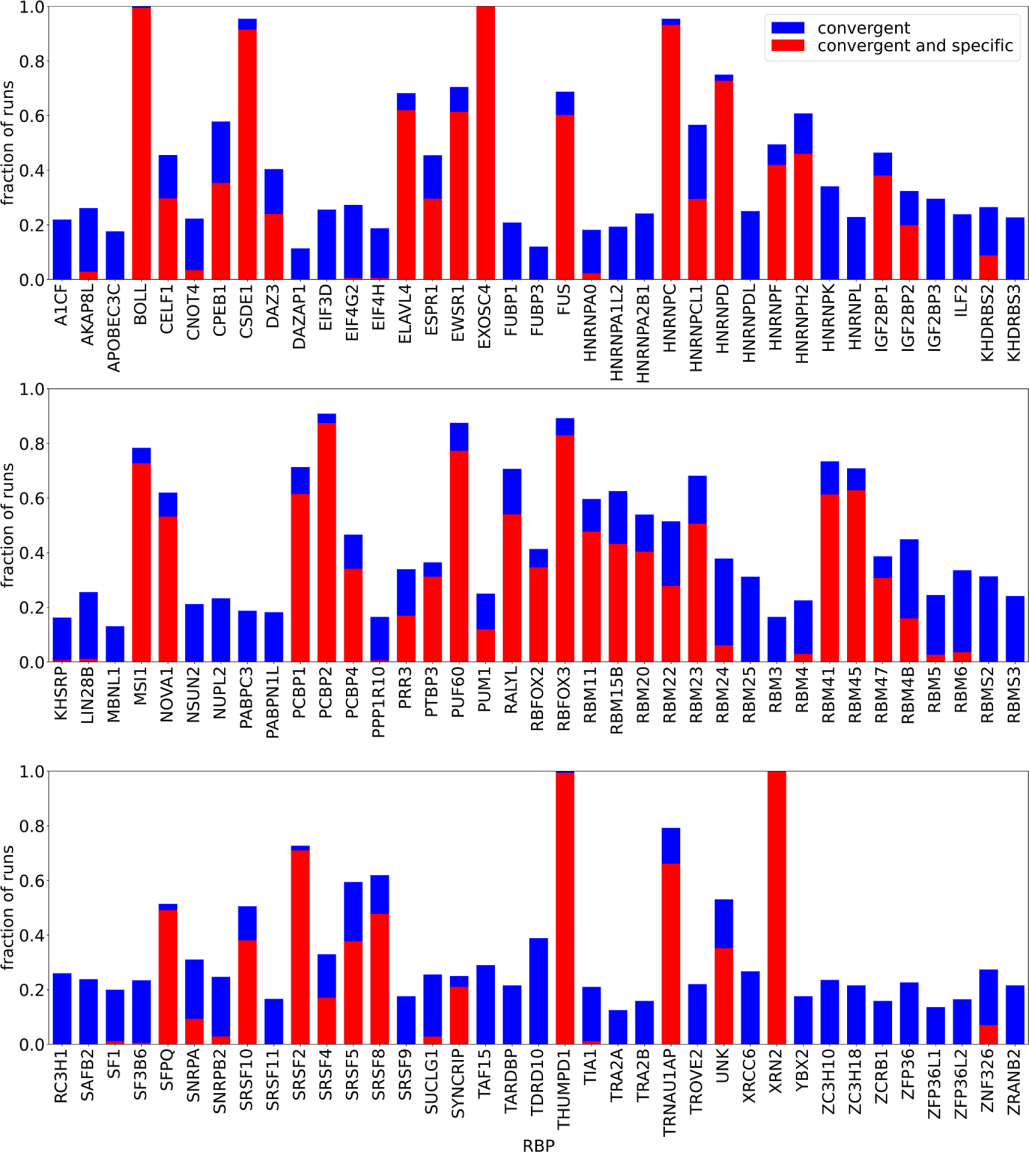
Summary of fraction of convergent and specific outcomes of 16 runs with random initializations per $L_{w}\in 5,6,7,\ldots ,14,15$ . “Convergent” means that the final log-likelihood was larger than the initial one, “specific” means that $E_{0}<0$ , i.e. the non-specific binding term has a limited contribution to the overall energy of RBP-oligomer interaction. Some runs did not finish within the given maximum walltime, therefore the absolute numbers of runs was not exactly the same for all RBPs. All runs done for a given RBP, regardless of the $L_{w}$ are shown together.

We analyzed all RNA Bind’n Seq data available in ENCODE ^10^ for 111 RBPs. For each RBP, we investigated 11 different binding site lengths, $L_{w}=5,6,7,\ldots ,14,15$ nts, where the lower limit was chosen as 5nts in agreement with the literature, and the upper limit of 15 nts was determined by the available RAM. Moreover, as most reads are 20 nts long, a larger $L_{w}$ approaching this value would also not be warranted. For each of pair of RBP and $L_{w}$ , we set up 16 runs with randomly initialized parameters and 4 runs in which the initial PWM was set close to the reported consensus motif (from RBP Bind’n Seq data). ^10^ We generally used four CPUs with three hours of maximum walltime per RBP, and eight CPUs when necessary (e.g. because the read length was larger than 20 nts. For the overwhelming majority of runs we found these resources to be sufficient, though a few runs did not complete during this time, thus not all RBPs had exactly the same number of finished runs. Figure 1 gives an overview of the run statistics. As a local minimum was not found in all randomly initialized runs, the figure shows the fraction of “convergent” runs, i.e. runs in which the final log-likelihood was larger than the initial one. In addition, we observed that even convergent runs were sometimes dominated by the unspecific binding term $e^{E_{0}}$ . Since in this case the inferred PWM is not meaningful as it does not explain the data as well as the sequence-unspecific term, we report the “specific” cases, where $E_{0}<0$ . As shown in Figure 1 all RBPs with a large fraction ( >0.4 ) of convergent runs, led to the recovery of a specific motif. In contrast, RBPs with a low fraction ( <0.3 ) of convergent runs had solutions dominated by unspecific binding.

**Figure 2.**
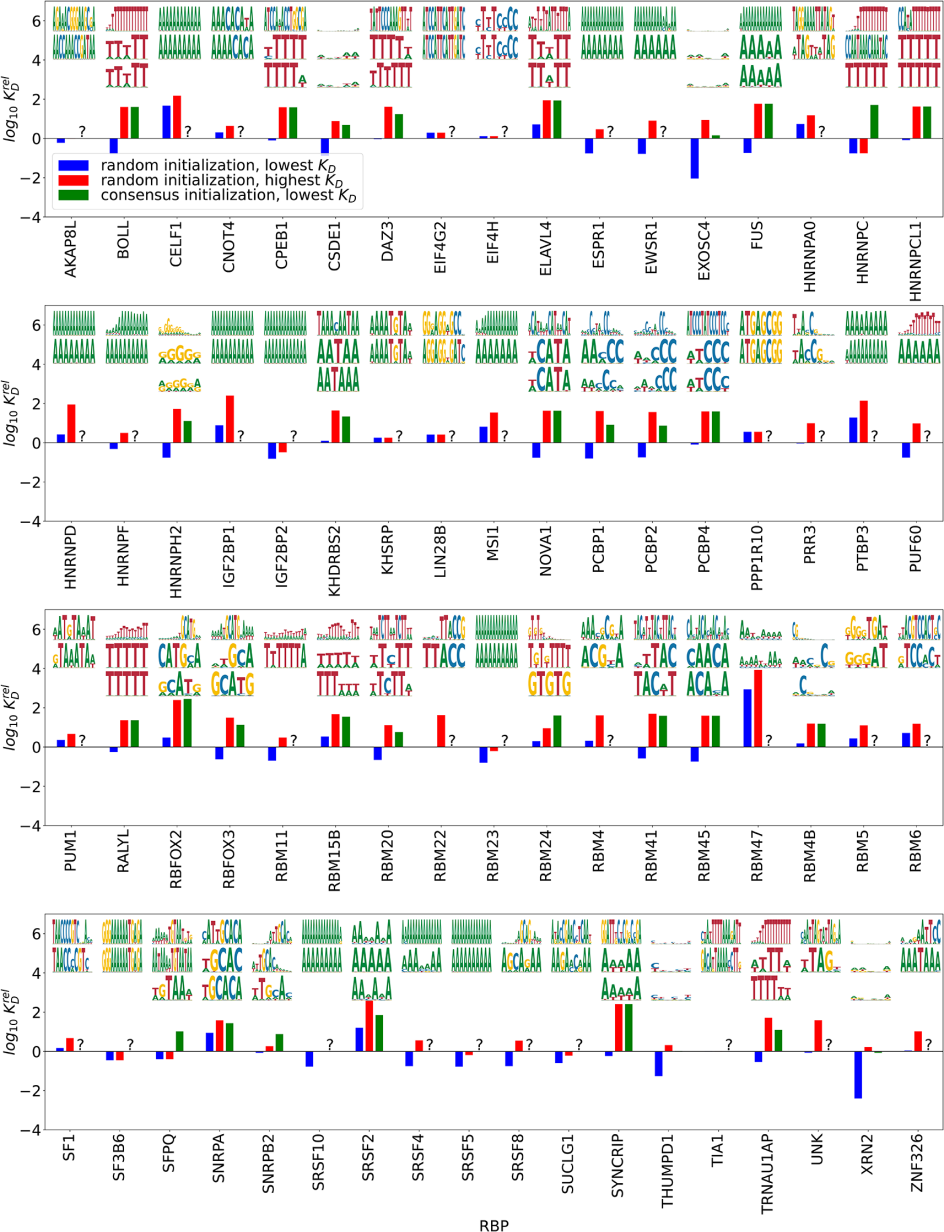
Inferred RBP binding specificities. $K_{D}$ ’s are relative to an unpolarized PWM of length 5 with no unspecific binding. Top/middle: highest/lowest affinity motifs (lowest/highest relative $K_{D}$ ) from random initialization, bottom: highest affinity motif from initializations with the consensus motif.

Following eq. (2.9), we calculated the dissociation constants $K_{D}$ s for all convergent and specific outcomes of the EM procedure. Dissociation constants were given relative to a PWM of length 5 , with equal probabilities for all four nucleotides ( 0.25 ) disregarding unspecific binding i.e. $E_{0}\to -\infty$ . This allows us to compare the binding strength of different motifs for an RBP with each other and also of motifs for different RBPs, as done in Figure 2. Below we highlight the motif and relative $K_{D}$ s for the motifs with the lowest and highest $K_{D}$ from random initialization, as well as the motif with the lowest $K_{D}$ obtained by initializing with the consensus PWM provided for the respective RBP by Ref. 10. As a first plausibility check, we find that longer motifs tend to have lower $K_{D}$ s, i.e. higher binding affinities, which is expected due to the larger number of bonds the larger binding sites can form with the RBP. We found convergent and specific results in 82 of 111 cases. Comparing the lowest $K_{D}$ motifs to the ones obtained by starting from the motifs found by kmer-enrichment analysis, ^10^ we found that the consensus motif is contained in or differs in at most one position in 48 cases. 19 of the highest affinity motifs did not contain the ENCODE consensus; in 17 of these cases our algorithm found a less complex motif (mostly poly(A)), while in two cases (for CPEB1 and EIF4H) the motifs found by our algorithm are more complex. In 14 cases, the motif found by our algorithm appeared related to the consensus, but it was less polarized or differed from consensus in more than one position. For one RBP, PTBP3, no motif was reported based on RNA Bind’n-Seq data, while we found a convergent and specific poly(A) motif. The seemingly large bias towards poly(A) in case of no other motif found is consistent with adenine being the most prominent nucleotide in all of the investigated libraries.

Below we assess the correspondence of the results given by our model with prior knowledge for a few proteins that have been extensively studied. Examples where we found motifs consistent with prior literature are RBFOX2, NOVA1, PUM1, PUF60, ELAVL4, hnRNPC, and PCBP1. In contrast, for CELF1, EWSR1, hnRNPD, and hnRNPK we either did not obtain a convergent and specific result or the result was clearly different from the reported consensus. We also discuss CPEB1, EIF4H, and PTBP3 as cases where our model appears to deliver new information. Note that as we start our analysis from sequenced DNAs we use T in the sequence logos, though of course, the RNA oligonucleotides contained U’s.

### 4.1 RBFOX2

**Figure 3.**
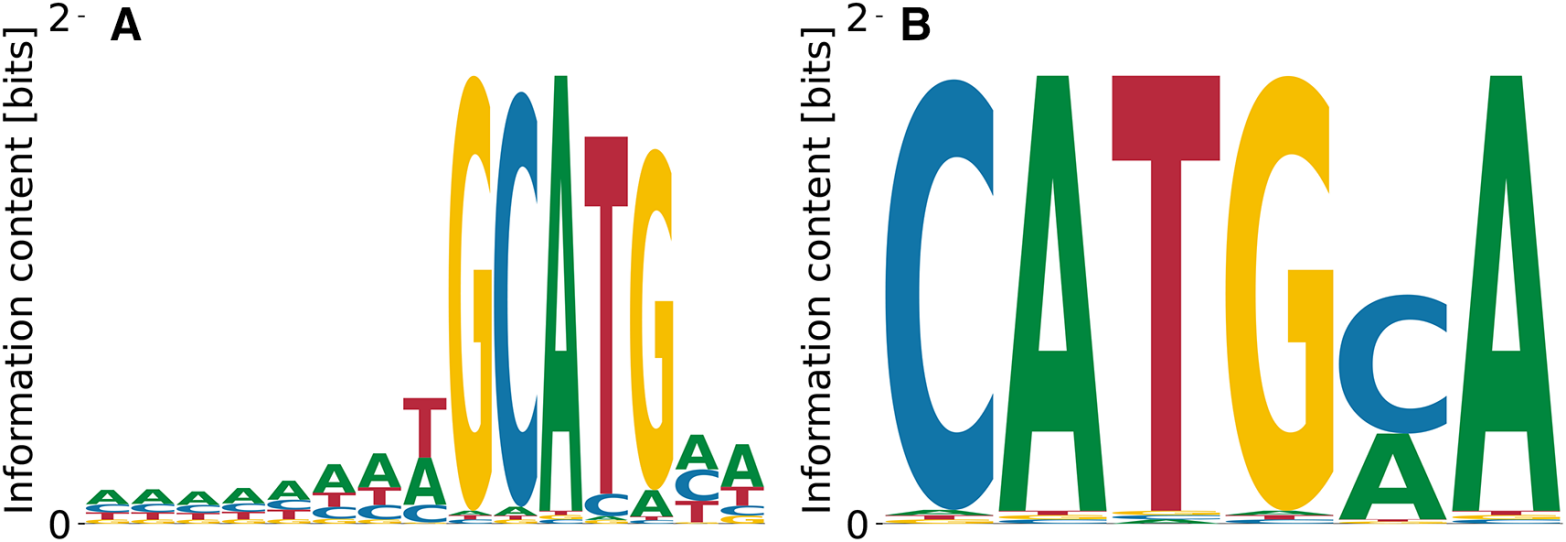
Lowest (A) and highest (B) *K*_D_ PWMs for RBFOX2 obtained from our model with random initialization. The highest affinity motif contains the known consensus GCATG, flanked by positions with low A/T bias. Both high and low affinity motifs share a CATG core.

RBFOX2 is a key regulator of alternative splicing ^16^ that was extensively studied with a variety of methods (e.g. Ref. 17). The RBFOX2 Bind’n Seq dataset ^10^ consists in nine libraries obtained using nine different protein concentrations and two protein-free control libraries, all containing reads of 50 nucleotides (nts) in length, including the adaptor. RBFOX2 is widely used to benchmark computational analysis methods (c.f. Ref. 18) and thus the corresponding dataset was carefully generated, to include multiple, high-quality libraries. Established techniques like kmer-enrichment analysis and the streaming-kmer-algorithm (SKA) predict a consensus 6mer TGCATG as the most prominent motif followed by other GCATG-containing 6mers. ^18^ Our results in Figure 3 reproduce the importance of the GCATG morif, but also the slight T bias at the preceding position, see Figure 3(A). Moreover, we find the subdominant PWM Figure 3(B) which shares a CATG core with the highest affinity motif, but over-emphasizes the A bias downstream. These results demonstrate that our algorithm identifies the known consensus for RBFOX2.

The log-values of relative $K_{D}$ ’s are in the range 0.48 - 2.39 , meaning that the two motifs shown in Figure 3(A) and (B) differ by ≈100 fold in their $K_{D}$ . The unspecific binding term in the run that yielded the motif from panel (A) was $E_{0}=-15.57$ , while the one from the panel (B) run was $E_{0}=-2.49$ . The overall log-likelihoods of the dataset were $-8.51\times 10^{9}$ vs. $-8.77\times 10^{9}$ , respectively. Thus, the higher affinity motif corresponds to a higher peak in the likelihood landscape, and accounts for a more specific interaction. The binding specificity of RBFOX2 in Figure 3(A) is similar to that of the closely related RBFOX3 (c.f. Figure 2).

### 4.2 NOVA1

**Figure 4.**
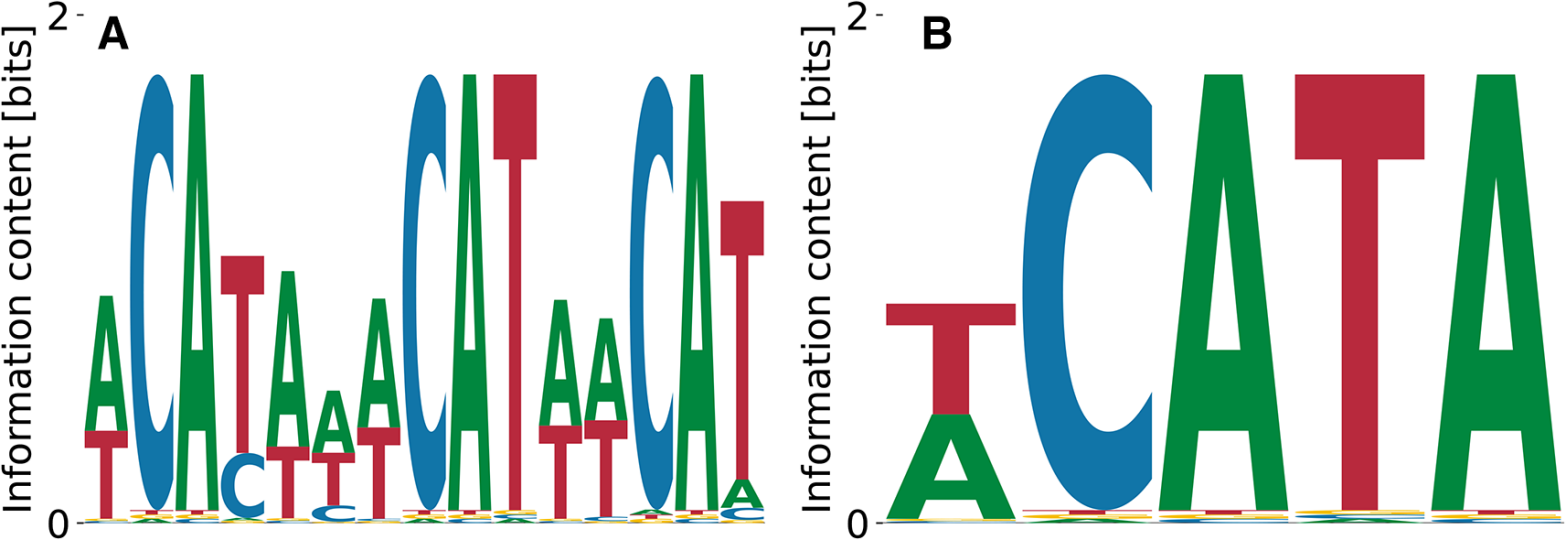
Lowest (A) and highest (B) *K*_D_ NOVA1 motifs obtained by our model with random initialization. (A) shows the characteristic triple CAT-repeat.

The neuro-oncological ventral antigen 1 (NOVA1) is a neuron-specific RBP ^19^ found by SELEX experiments to bind a triple CAT-repeat. ^20^ This is confirmed by the motif identified by our algorithm ( Figure 4). In contrast, the streaming kmer-enrichment analysis of RNA Bind’n Seq data in ENCODE predicted a single CAT. ^10^

Relative log- $K_{D}$ values ranged from −0.76 to 1.63 , the unspecific binding term was $E_{0}=-6.10$ for Figure 4(A) and $E_{0}=-2.61$ for Figure 4(B), and the log-likelihoods of the data were $-3.80\times 10^{9}$ and $-7.91\times 10^{9}$ , respectively.

### 4.3 PUM1

**Figure 5.**
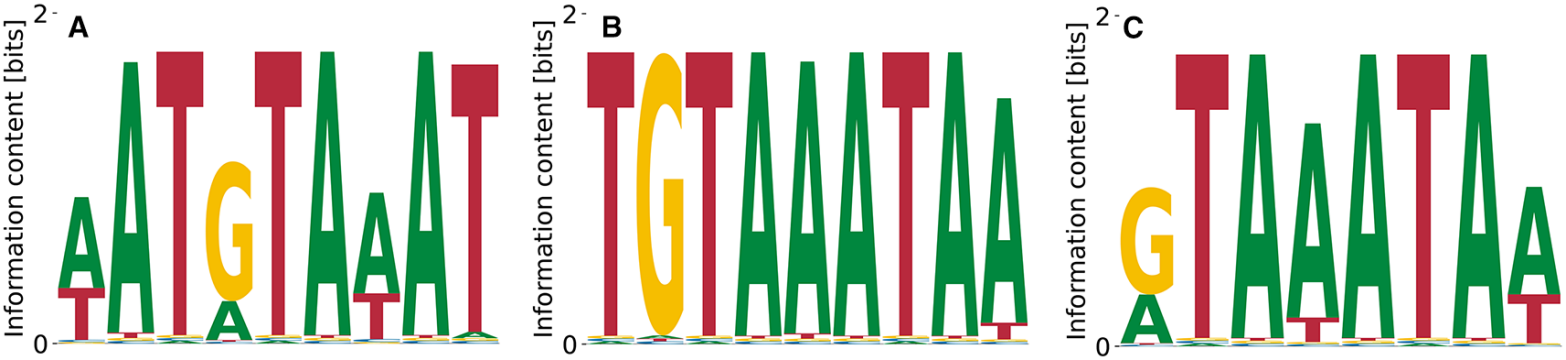
Lowest (A), intermediate (B), and highest (C) *K*_D_ PWMs for PUM1 obtained from our model with random initialization. The intermediate motif (B) represents the consensus. ^22^

Pumilio homolog 1 is a well-studied member of the PUF famility of proteins, which binds to 3’UTRs of a variety of mRNAs and regulates their translation. ^21^ While immunoprecipitation (IP) experiments found its binding specificity to be TGTAHATA, ^22^ SKA analysis of RNA Bind’n Seq yielded TGTAH without further context. ^10^ In contrast, we do find the full motif in the Bind’n Seq data ( Figure 5). All obtained motifs are related to each other via shifts and show only gradual differences at single positions, which also reflects in characteristic numbers ( $K_{D}$ , $E_{0}$ , and $\log P\left(D|M,E_{0}\right)$ ) being distributed only over a narrow range. The relative log-dissociation constants for Figure 5 are 0.36 , 0.36 , and 0.67 , where the former two differ by only 0.0004 . The corresponding unspecific binding strengths are −3.47 , −1.21 , and −2.58 . The log likelihoods of the data range from $-1.39\times 10^{9}$ to $-1.43\times 10^{9}$ .

### 4.4 PUF60

**Figure 6.**
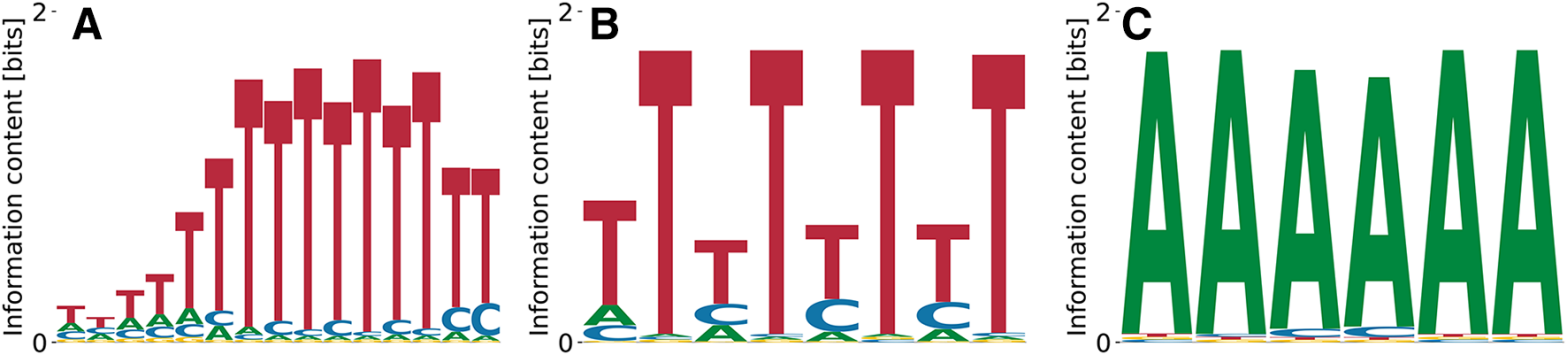
Lowest (A), intermediate (B) and highest (C) *K*_D_ PWMs for PUF60 obtained from our model with random initialization.

A representative from the same family of RBPs is the Poly(U)-binding splicing factor PUF60, ^23^ which regulates both transcription and translation. ^24^ Our algorithm finds the expected motif (poly(T)) as having the highest affinity Figure 6(A). The relative log- $K_{D}$ values for T-rich motifs are ≥−0.76 , with $-10.53\geq E_{0}≳-7.5$ and $\log P\left(D|M,E_{0}\right)\leq -2.81\times 10^{9}$ . Different variations (see Figure 6(B)) of the poly(T) motif are found, having in common the alternating pattern of very polarized – less polarized T (the latter blended with C). Poly(A)-containing motifs (c.f. Figure 6(C)) also appear at $\log K_{D}^{\mathrm{rel}}>-0.33$ and $E_{0}>-5.49$ , with $\log P\left(D|M,E_{0}\right)\leq -4.04\times 10^{9}$ . The motif with the highest dissociation constant obtained from random initializations is shown in Figure 6(C), has $\log K_{D}^{\mathrm{rel}}=0.98$ , and corresponds to $E_{0}=-0.64$ and $\log P\left(D|M,E_{0}\right)=-5.68\times 10^{9}$ .

### 4.5 ELAVL4

**Figure 7.**
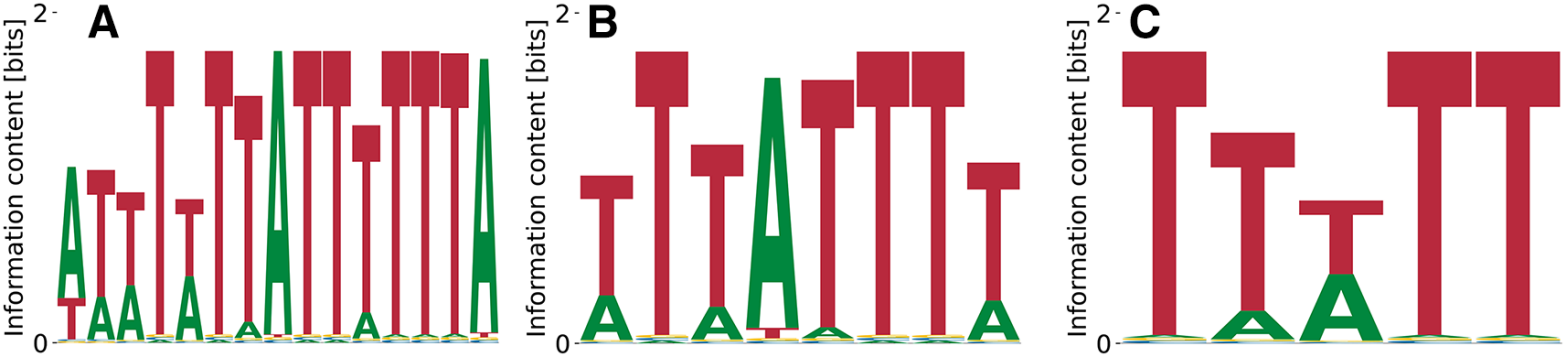
Lowest (A), intermediate (B), and highest (C) *K*_D_ PWMs for ELVAL4 obtained from our model with random initialization.

The ELAV-like protein 4 (ELAVL4) is an RBP that is exclusively expressed in neurons. ^25^ It binds A/T-rich elements according to genome-wide IP experiments, ^26^^,^^27^ a pattern that we also observe in PWMs from our model (see Figure 7). Relative log- $K_{D}$ ’s range from 0.71 to 1.94 , unspecific binding strength from $E_{0}=-3.46$ to $E_{0}=-2.24$ , and the log-likelihood of the data from $-1.95\times 10^{9}$ to $-2.07\times 10^{9}$ .

### 4.6 HNRNPC

**Figure 8.**
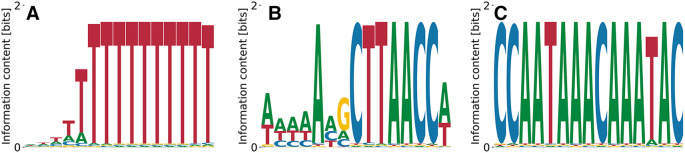
Lowest (A), intermediate (B), and highest (C) *K*_D_ PWMs for hnRNPC obtained from our model with random initialization.

Turning towards another large familiy of RBPs, the heterogeneous nuclear ribonucleoproteins (hnRNPs), we highlight hnRNPC, ^28^ a protein that is involved pre-mRNA processing. ^29^ It is known to bind to poly(U) motifs, typically pentamers, found in both SKA analysis of RNA Bind’n Seq data ^10^ and in electrophoretic mobility shift assays. ^30^ Our model does recover the dominant poly(T) motif at $\log K_{D}^{\mathrm{rel}}=-0.76$ , $E_{0}=-8.54$ , and $\log P\left(D|M,E_{0}\right)=-2.09\times 10^{9}$ . Mildly subdominant motifs are A-rich and less repetitive ( Figure 8(B), (C)) with $\log K_{D}^{\mathrm{rel}}=-0.76$ , $E_{0}=-7.67$ , and $\log P\left(D|M,E_{0}\right)=-2.09\times 10^{9}$ , as well as $\log K_{D}^{\mathrm{rel}}=-1.01$ , $E_{0}=-8.54$ , and $\log P\left(D|M,E_{0}\right)=-2.09\times 10^{9}$ .

**Figure 9.**
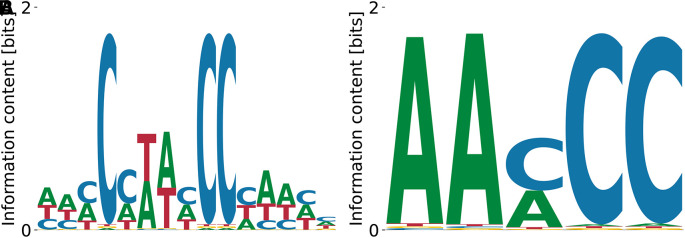
Lowest (A) and highest (B) *K*_D_ PWMs for PCBP1 obtained from our model with random initialization.

### 4.7 PCBP1

As a last example of RBPs of which we predict a motif that agrees with prior data, we highlight the poly (rC)-binding protein 1 (PCBP1). ^31^ Its binding specificity consists of a few very polarized C’s linked by A/T-enriched sequence. ^32^ This pattern is clearly reproduced by our highest affinity motif in Figure (9)(A) ( $\log K_{D}^{\mathrm{rel}}=-0.81$ ) with $E_{0}=-13.26$ and $\log P\left(D|M,E_{0}\right)=-2.99\times 10^{9}$ . The lowest affinity motif ( Figure 9(B)) that random PWM-initialization yielded had $\log K_{D}^{\mathrm{rel}}=1.62$ , $E_{0}=-1.49$ , $\log P\left(D|M,E_{0}\right)=-6.07\times 10^{9}$ .

### 4.8 CELF1

CELF1 is an RBP of the CUG-binding CELF family, ^33^^,^^34^ that participates in multiple steps of post-transcriptional processing of RNAs, including splicing, translation and decay. ^35^ CELF1 requires UGU motifs for high-affinity interaction with RNAs. ^36^ The corresponding Bind’n Seq dataset ^10^ consists of libraries generated for seven different RBP concentrations, each containing $∼2\times 10^{7}$ reads of $L_{S}=40$ .

**Figure 10.**
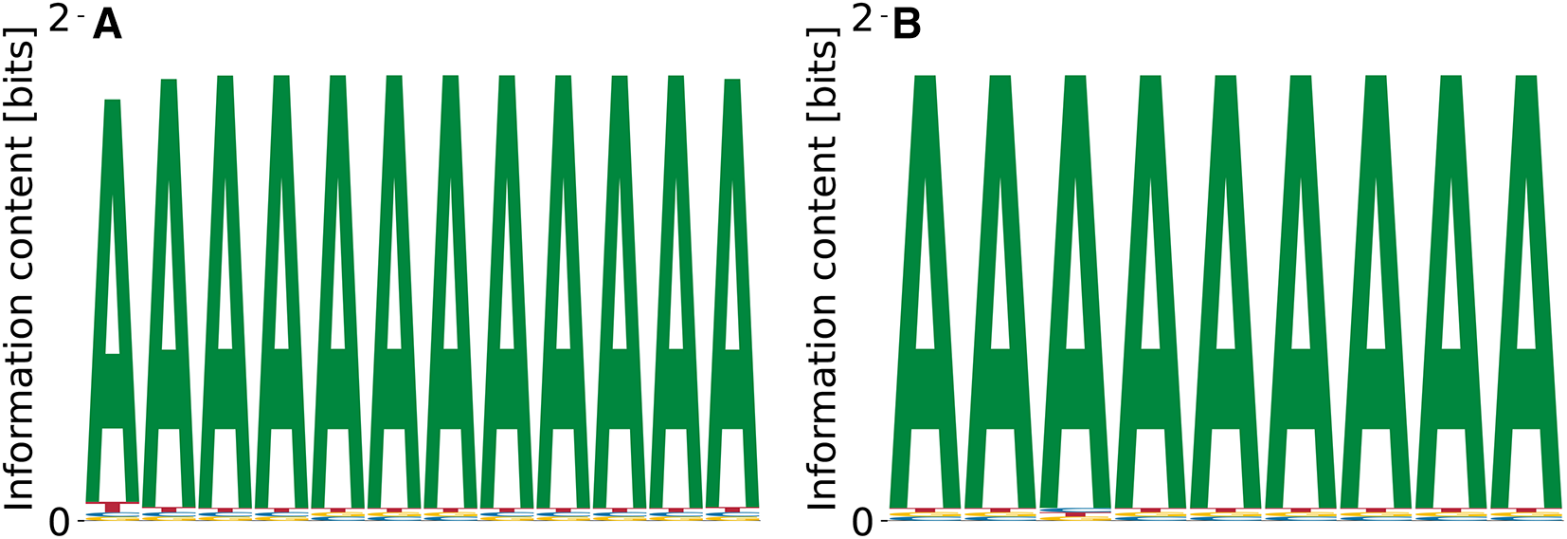
Lowest (A) and highest (B) *K*_D_ PWMs for CELF1 predicted by our model from random initialization. Enrichment analysis suggests a prominent TGT as consensus, which our model cannot recover, even when initializing with NTGTN .

Since 16 runs with completely random PWM intialization for $L_{w}=5,6,\ldots ,14,15$ did not yield any local optimum of the probability landscape we decided to test whether the biased initialization of the PWM with the known motif (TGT), which was found as as enriched 3-mer in RNA Bind’n Seq ^10^ enables the recovery of longer motifs. However, our model predicted only poly(A) stretches as convergent optima of the probability landscape, see Figure 10(A). The relative log-dissociation constants were $1.67≳\log K_{D}^{\mathrm{rel}}≳2.17$ , unspecific binding was characterized by $-4.0≳E_{0}≳-0.75$ , and the likelihood of the daat was between $\log P\left(D|M,E_{0}\right)-8.41\times 10^{9}$ and $-8.48\times 10^{9}$ .

### 4.9 EWSR1

**Figure 11.**
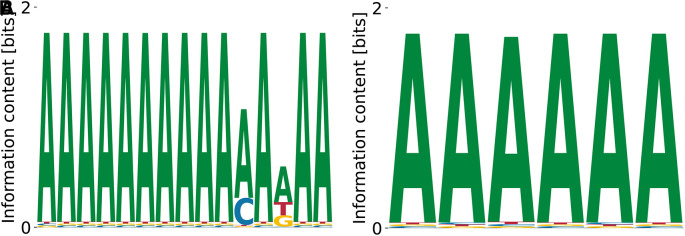
Lowest (A) and highest (B) *K*_D_ PWMs for EWSR1 predicted by our model from random initialization.

EWSR1, or Ewing’s sarcoma protein, forms fusions with a number of other proteins and serves as a transcriptional activator in human solid tumors like Ewing’s sarcoma and malignant melanoma. ^37^ Information about its binding specificity is scarce, while SKA analysis of RNA Bind’n Seq data predicts G-rich elements. ^10^ Our investigation lead to different varieties of poly(A) motifs ( Figure 11(A) and (B)), which vary in relative dissociation constants $-0.79\leq \log K_{D}^{\mathrm{rel}}\leq 0.91$ in log- $K_{D}$ , unspecific binding energy $-4.18\leq E_{0}\leq -0.55$ , and data likelihoods $-2.2\times 10^{9}\geq \log P\left(D|M,E_{0}\right)\geq -4.50\times 10^{9}$ .

### 4.10 HNRNPD

**Figure 12.**
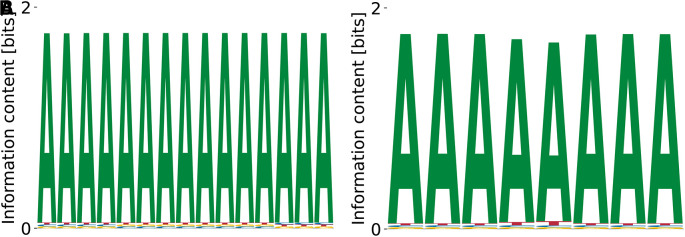
Lowest (A) and highest (B) *K*_D_ PWMs for hnRNPD predicted by our model from random initialization. Enrichment analysis suggests a quite unpolar A/T-rich binding domain. Both motifs, (A) and (B), show A as the dominant nucleotide, followed by T at each position.

Within the class of heterogeneous ribonucleoproteins (hnRNPs), hnRNPD (also known as AUF1) is a well-known A/U-rich element RNA binding protein with important role in RNA decay. ^38^ HNRNPD has been reported to bind clusters of AUUUA elements. ^38^ The ENCODE-database ^10^ lists AUAAU as another possible binding site for hnRNPD. We find poly(A) stretches of different lengths as convergent and specific binding motifs (see Figure 12). While A is the dominating nucleotide at every position, it is followed consistently by T, which fits the reported A/U-rich binding domains in the literature. We find unspecific binding terms in the range $-7.56\geq E_{0}\geq -2.48$ , where Figure 12(B) corresponds to the highest affinity motif ( Figure 12(A)) for which $E_{0}=-3.17$ . The two motifs shown in Figure 12 come from a range of dissociation constants $0.42\leq \log K_{D}^{\mathrm{rel}}\leq 1.95$ and data likelihoods $-2.23\times 10^{9}\geq \log P\left(D|M,E_{0}\right)\geq -2.30\times 10^{9}$ . Direct initialization with the kmer-enrichment consensus ATAAT did not lead to any convergent and specific result.

### 4.11 HNRNPK

We were also interested in determining whether we can recover G/C-rich binding motifs from the data and therefore applied the model to heterogeneous nuclear ribonucleoprotein K (hnRNPK), a member of the poly(C) binding family of proteins. ^39^ We could only recover one of the two consensus motifs reported in the ENCODE analysis of these data (GCCCA, from SKA) ^10^ when specifically initializing the PWM with this motif. The second reported motif, with the CACGC consensus, could not be found by our algorithm even when the PWM was initialized with the motif itself and even when sequences containing the first motif were eliminated, indicating that this motif does not correspond to a local maximum of the likelihood function. We did not find any PWMs of $L_{w}>5$ in this data set, whether we used random initialization or shorter motif-guided initialization.

### 4.12 CPEB1

**Figure 13.**
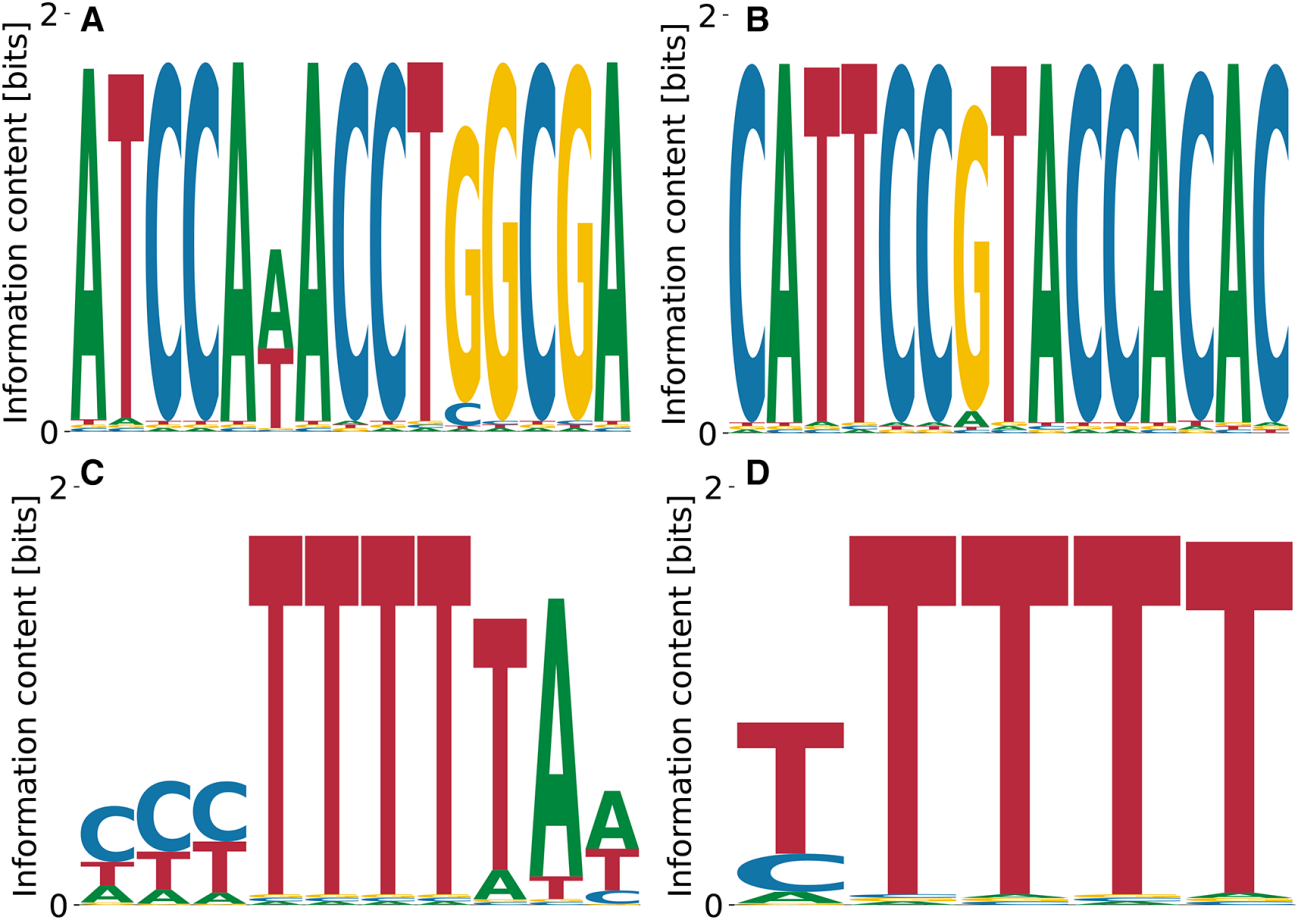
Lowest (A), intermediate (B) and (C), and highest (D) *K*_D_ PWMs for CPEB1 predicted by our model from random initialization. Although the consensus binding motif is recovered in (C) and (D), we predict (A) and (B) to be stronger binding specificities.

The cytoplasmic polyadenylation element-binding protein 1 (CPEB1) ^40^ serves as a translational regulator by binding specific U-rich sequences in 3’UTRs inducing cytoplasmic adenylation. The streaming kmer algorithm predicts a poly(T) motif from RNA Bind’n Seq data, which is what we recover as the lowest affinity motif in Figure 13(D) with $E_{0}=-2.39$ , $\log P\left(D|M,E_{0}\right)=-3.30\times 10^{9}$ , and $\log K_{D}^{\mathrm{rel}}=1.59$ . The penta(T) is also part of a higher affinity motif (see Figure 13(C)) with $E_{0}=-5.90$ , $\log P\left(D|M,E_{0}\right)=2.92\times 10^{9}$ , and $\log K_{D}^{\mathrm{rel}}=0.00$ . More complex motifs with higher affinity, but less specificity are displayed in Figure 13(A) with $E_{0}=-2.05$ , $\log P\left(D|M,E_{0}\right)=-2.56\times 10^{9}$ , and $\log K_{D}^{\mathrm{rel}}=-0.10$ and in Figure 13(B) with $E_{0}=-1.32$ , $\log P\left(D|M,E_{0}\right)=-2.56\times 10^{9}$ , and $\log K_{D}^{\mathrm{rel}}=-0.10$ . While the difference in affinity between the first three motifs shown in Figure 13(A-C) is negligible, while the penta(T) motif alone ( Figure 13(D) differs from the highest affinity one by ≈50 -fold, which is quite substantial.

### 4.13 EIF4H

**Figure 14.**
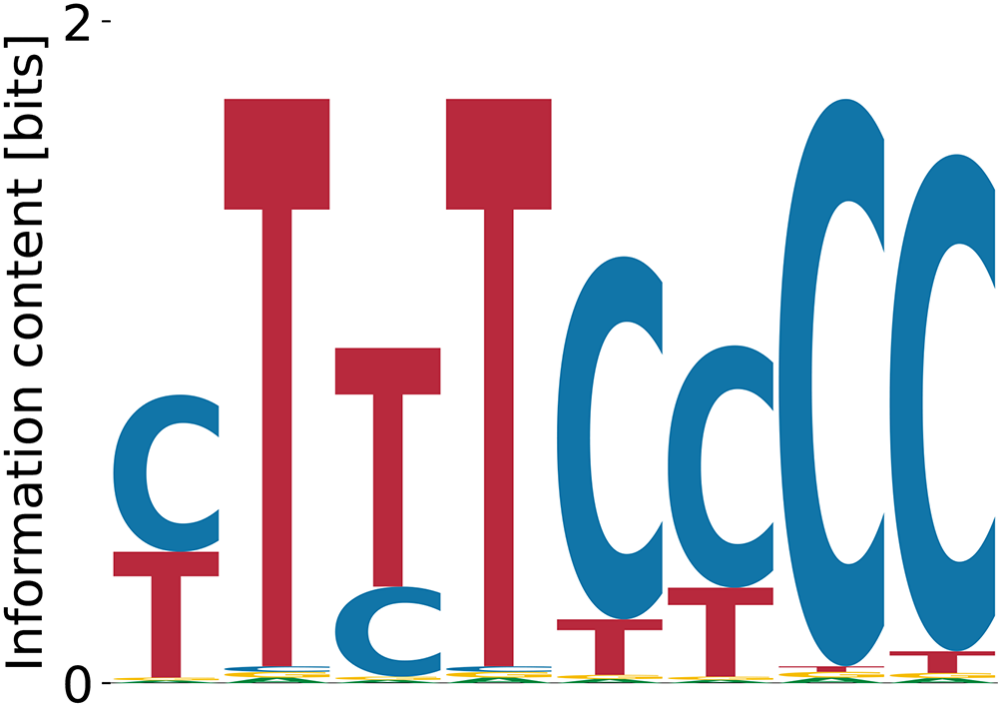
Single convergent and specific PWM for EIF4H predicted by our model from random initialization.

As a representative of the family of eukaryotic translation initiation factors, we highlight the eukaryotic translation initiation factor 4H (EIF4H). ^41^ SKA enrichment studies of RNA Bind’n Seq data predict a poly(G) motif for this protein, ^10^ but we did not find corroborating data obtained with other approaches. Our model identified only one convergent and specific motif shown in Figure 14, which is pyrimidine-rich. The relative log- $K_{D}$ is relatively high, $\log K_{D}^{\mathrm{rel}}=0.11$ , as is the contribution of unspecific binding, $E_{0}=-0.67$ . $\log P\left(D|M,E_{0}\right)=-7.44\times 10^{9}$ .

### 4.14 PTBP3

**Figure 15.**
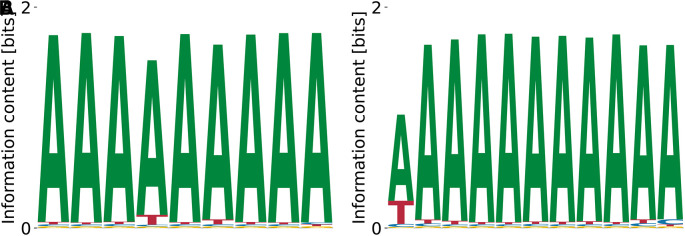
Lowest (A) and highest (B) *K*_D_ PWMs for PTBP3 predicted by our model from random initialization.

Polypyrimidine tract-binding protein 3 (PTBP3) is involved in the regulation of numerous steps of protein production, i.e. splicing, alternative 3’ end processing, mRNA stability and RNA localization. ^42^ Data on PTBP3 binding specificity is lacking, and enriched kmer was reported by the SKA from RNA Bind’n Seq data. ^10^ Our model predicts different variations of poly(A), see Figure 15. The predicted binding specificities with the lowest $K_{D}$ features $E_{0}=-3.02$ , $\log K_{D}^{\mathrm{rel}}=1.28$ , and $\log P\left(D|M,E_{0}\right)=-1.15\times 10^{9}$ . The motif corresponding to the highest $K_{D}$ had $E_{0}=-3.86$ , $\log K_{D}^{\mathrm{rel}}=2.14$ , and $\log P\left(D|M,E_{0}\right)=-1.12\times 10^{9}$ . Thus, compared to other RBPs (c.f. Figure 14), the specificity of PTBP3 remains to be better defined.

### 4.15 Other RBPs

There are other proteins covered in the Bind’n Seq data ^10^ whose specificity was studied before. For example, we analyzed the data corresponding to MBNL1, ^43^ hnRNPL, ^44^ FUS, ^45^ TAF15. ^46^

**Figure 16.**
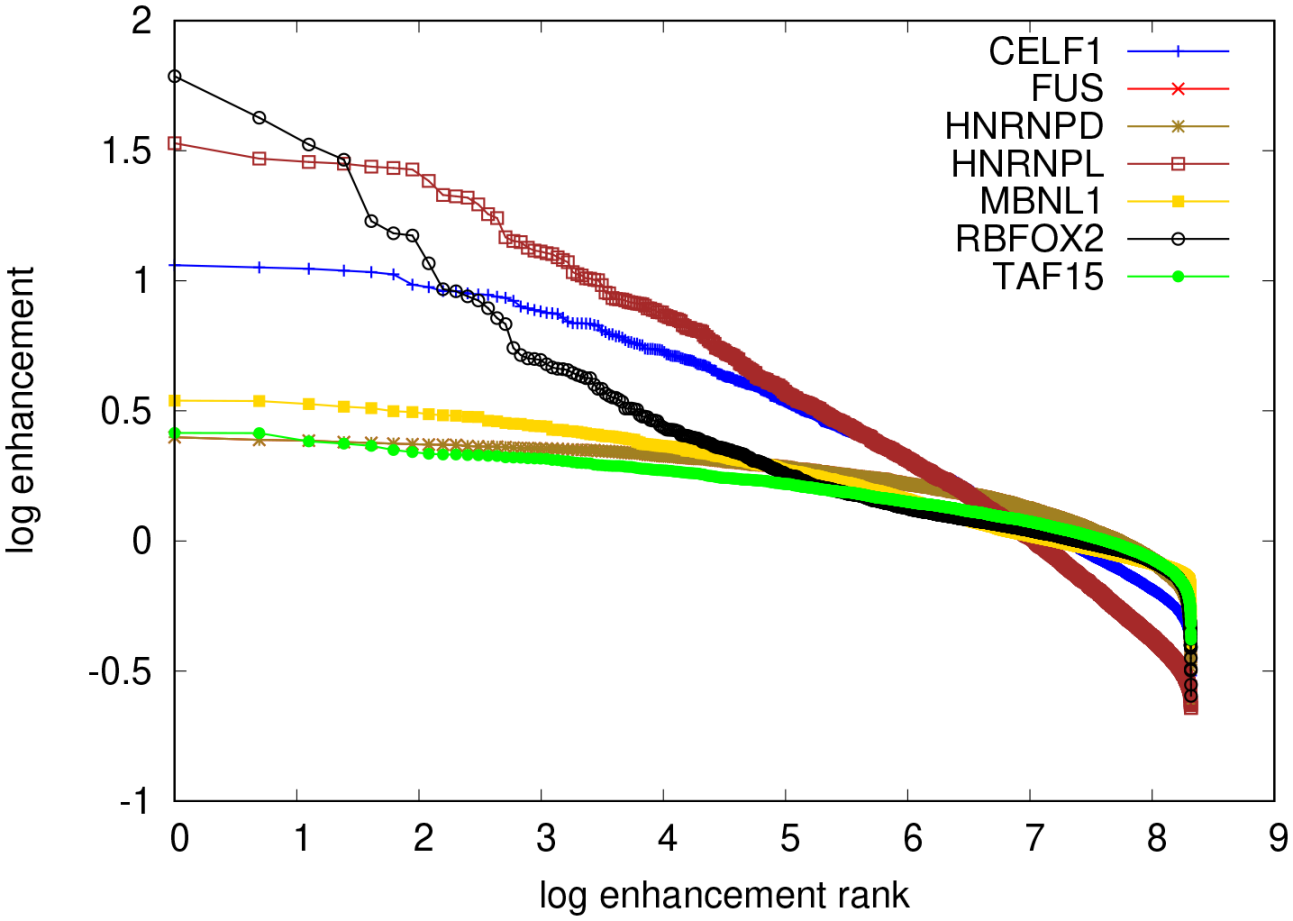
Logarithmic (base e ) enrichment (count in foreground reads normalized by count in background) for all $4^{6}=4096$ possible 6mers in all datasets (corresponding to different RBPs), ranked by enrichment. The top most enriched motifs are for RBFOX2: TGCATG, FUS: GCGCGC, hnRNPL: CACACA, MBNL1: GCTGCT, TAF15: GGGGGG. All except the RBFOX2 motif are repeats of shorter oligomers.

Random and consensus intialization did result in convergent and specific binding specificities for FUS, however, these were poly(A) motifs which do not agree with the GGUG consensus from SELEX experiments. ^47^ For the other threee proteins our model did not deliver any convergent results, even when the PWM was directly initialized with the expected consensus motif. This indicates that the enrichment did not work equally well for all the RBPs studied with the Bind’n Seq method or that our method does not identify well motifs that are very short ( ∼2 nts, observed in the SKA enrichment analysis). In this analysis, kmer enrichments are computed by counting the number of occurrences of every possible kmer in the foreground samples (RBP concentration ≠0 ) and in the background samples (RBP concentration =0 ), and finally normalizing the foreground abundances by the background to extract the respective enrichment. The higher the enrichment of a given kmer, the higher the likelihood of it being bound by the RBP used in the experiment is thought to be. We computed these enrichments for 6mers, as done in the ENCODE studies. The results, shown in Figure 16 indicate that only RBFOX2 has a very clear hierarchy of top enriched 6mers, while the other investigated RBPs show a much flatter hierarchy of motif enrichments. An analysis of the Levenshtein distance of these motifs showed no clear difference in the pattern of distances among the leading motifs across the investigated RBPs. This suggests that these motifs correspond to many local minima of comparable depth, which precludes our algorithm finding clear PWMs representing the binding sites. Conversely, it becomes unclear whether the specificity of these RBPs would be well represented as weight matrices, or whether another model, for e.g. clusters of short, degenerate motifs may better represent the specificity of these RBPs.

## 5. Conclusion

We constructed a thermodynamic model that can be used to infer characteristic position weight matrices for the binding domains of RBPs from data obtained from affinity-based enrichment of oligonucleotides. Since we directly model the RBP-binding specificity as PWMs, our method bypasses arbitrary choices in the alignment of kmers found to be individually enriched in the data. We evaluate our model on 111 RNA Bind’n Seq data sets in the public domain, ^10^ finding convergent and specific motifs for 82 RBPs. For 48 of these there is complete agreement with previously reported motifs, 14 partly agree, 19 disagree, and one did not have a reported motif (PTBP3). In most cases of disagreement, our model predicts the binding element to be, in fact, poly(A), while in two cases (CPEB1 and EIF4H), we predict more complex motifs than previously reported.

Many of the RBPs for which we did not recover a PWM tend to be more degenerate motif than RBPs for which some motif emerged. E.g. ESRP1, EWSR1, FUS and other proteins have been shown to bind G-repeats, which are relatively rare in the Bind’n Seq libraries. It is likely that to identify such motifs it is crucially important that the background model is accurate. How to best construct this model remains to be determined in future work. In addition, it is possible that the binding sites of these proteins are not contiguous, linear motifs, but rather contain variable length spacers of form structures such as G-quadruplexes. It will thus be important to explore other types of models in the future, e.g. models that allow more flexible spacing of RBP contact points on RNAs in the future.

## Data Availability

DOI’s are doi:10.17989/ < experiment identifier > .

## Software availability

Source code available from: https://git.scicore.unibas.ch/zavolan_group/pipelines/bind-n-seq-pwms ^15^

Archived source code and results of runs at time of publication of version 2: https://doi.org/10.5281/zenodo.11072860 ^48^

License: MIT
