# Supplementary material for: On the limits of inferring biophysical parameters of RBP-RNA interactions from in vitro RNA Bind’n Seq data
Source: F1000Res. 2024 May 29;12:742. Originally published 2023 Jun 26. [Version 2] doi: 10.12688/f1000research.135164.2 (PMC11269977; doi:10.12688/f1000research.135164.2)
Supplement: Peer review history

# Reviewer report: Reviewer response for version 2

**DOI:** 10.5256/f1000research.165610.r284353

**Published:** 2024-07-24

**Authors:** Jun Zhang

**Affiliations:** 1 Texas Tech University Health Science Center El Paso (TTUHSCEP), El Paso, USA

The revision has addressed the issues in the first version.

Is the rationale for developing the new method (or application) clearly explained?

Partly

Is the description of the method technically sound?

Yes

Are the conclusions about the method and its performance adequately supported by the findings presented in the article?

Partly

If any results are presented, are all the source data underlying the results available to ensure full reproducibility?

No source data required

Are sufficient details provided to allow replication of the method development and its use by others?

No

Reviewer Expertise:

RNA-binding protein, structural biology.

I confirm that I have read this submission and believe that I have an appropriate level of expertise to confirm that it is of an acceptable scientific standard.

# Reviewer report: Reviewer response for version 2

**DOI:** 10.5256/f1000research.165610.r299542

**Published:** 2024-07-22

**Authors:** Andreas Schlundt

**Affiliations:** 1 Institute for Biochemistry, University of Greifswald, Greifswald, Germany

This manuscript by Schlusser and Zavolan provides a bioinformatic approach to infer PWM representations in motifs targeted by RNA-binding proteins. It uses available RBnS data from the ENCODE database. Its suggested strength and improvement over the canonical way to infer consensus motifs in RBnS is that it bypasses the prior definition of kmers for the alignment procedure of enriched sequences.

 As a result the authors present convergent and specific motifs for 82 RBPs (out of 111 in the database). For 48 of these there is complete and for 14 partial agreement with previously reported motifs considering the RBnS database as the correct reference.

 The current manuscript version had undergone a prior round of revision with visible improvement. Although I am not an expert in the bioinformatic background I can nevertheless clearly see the (functionally relevant) motivation of the approach and highly appreciate it in light of the need to try to exploit the in-depth information of HTS-derived motifs of RBPs beyond the current standards. Still, I feel the approach misses to address or at least discuss the most obvious challenge (which could on the longer run be implemented?): the majority of RBPs uses more than one domain for interacting with RNA and individual domains may contribute with orders of magnitude difference in affinity to the combined specific target motif, while only this combined motif is the functionally relevant specific one. While this is an ongoing challenge in the interpretation of all CLIP and RBnS data, I wonder how much the herein presented may address this issue a bit more realistic.

 Together with this, here are my concrete points I suggest to address and clarify:

 1) The authors themselves mention the possible role of spaced elements targeted by multiple domains within one RBP. Similar to RBnS, a proper analysis towards this direction is challenging, but could e.g. include the consideration of multiple motifs. Have the authors thought about this? I suggest a bit more discussion into this direction. Mixed motifs with weighted contributions from relative affinities of domains will remain a major point to address in the future.

 2) In line with above: Considering multiple binding sites of an RBP on an oligo may mean more than one RBP binding to it OR multiple domains of one RBP binding to one combined element. I may not have fully understood how the estimation of Kd values according to the formula (2.6) may take this into consideration?

 3) Why is (page 5) the assumption that RBP binding is usually of low affinity in order to simplify the equation? Many RBPs bind in the low nM range, which is pretty strong, be it specific or not.

 4) Is there any particular way to treat motifs that are apparently part of RNA structure (Rc3h1)? This should be highly converging and specific reg. the central 5mer, but likely not in a larger context (i.e. the stem beneath the 3-5 nt loop). There may be less such structured motifs according to our expectations, but on the other there could be more of them hidden the RBnS data and we just neglect their fold context.

 5) For the les converging/specific RBPs: is there a correlation to their amount of domains present? And adding to that: seeing the motifs shown in Fig. 2, some seem to show large differences between the low vs. high K _D_. Could this correlate with the number of RBDs in the RBP, such that the affine ones come from the strong-binding domains and the other one from the subordinate domains? And: could also the occurrence of polyA relate to the number of domains and thus too many parallel motifs (e.g. for the IGF2BPs)? Apparently exactly those ones would need the definition of a motif cluster, while current approaches merely provide a weighted motif, which in the end is not precisely the right one for any of the six domains.

 6) Have the authors specifically taken into consideration the different concentrations of RBPs in the RBnS data? Esp. when there are multiple concentrations tested? Sorry if I have missed this.

 7) Could the authors in the end provide a somewhat fair discussion of how their inferred motifs now should be seen relative to RBnS motifs?! Do they suggest to question RBnS logos (which in the end still could be "wrong”) or do they just claim to have provided a faster and more efficient, user-friendly way to derive logos from RBnS data?

Is the rationale for developing the new method (or application) clearly explained?

Yes

Is the description of the method technically sound?

Yes

Are the conclusions about the method and its performance adequately supported by the findings presented in the article?

Partly

If any results are presented, are all the source data underlying the results available to ensure full reproducibility?

Yes

Are sufficient details provided to allow replication of the method development and its use by others?

Partly

Reviewer Expertise:

Structural biochemistry/biophysics of multi-domain RNA-binding proteins and their target RNAs centered around solution methods NMR and SAXS

I confirm that I have read this submission and believe that I have an appropriate level of expertise to confirm that it is of an acceptable scientific standard, however I have significant reservations, as outlined above.

# Reviewer report: Reviewer response for version 2

**DOI:** 10.5256/f1000research.165610.r299537

**Published:** 2024-07-09

**Authors:** Junbai Wang

**Affiliations:** 1 University of Oslo, Campus AHUS/Oslo, Norway

Manuscript by Schlusser N and Zavolan M describe a biophysical model for inferring motifs of RNA-binding proteins. They describe the derivation of LL function to the proposed EM algorithm for predicting enriched RNA binding motifs in sequences. The method was tested on RNA Bind’n Seq data from Encode with success on 7 RNA-binding proteins but failed on the other 4 RNA-binding proteins mentioned in the main text. In total, authors tested the method on 111 RNA-binding datasets, 82 of them obtained results where 48 with good agreement but 34 with either marginal or poor agreement. In summary, the success rate of this new method on 111 datasets is only around 43%, which is very low when compared to many public tools in DNA/RNA motif analysis. There are three major problems in the manuscript:

1. The theory behind their proposed biophysical model for motif finding is very unclear to me. Authors claim that their method is based on Boltzmann machine, which has been used many decades in protein-DNA interaction theory such as a classical paper in biophysics published in 1987 Berg OG et.al.,(ref 1 ), several applications of it in DNA sequences analysis in Djordjevic M et.al., 2003 (Ref 2) and Foat BC et.al., 2006 (Ref 3), and new development in more advanced Fermi-Dirac form of protein-DNA interactions by considering protein concentration with a Bayesian solution Wang J et.al., 2009 (Ref 4) Yang M et.al., 2023 (Ref 5) for DNA motif finding. From my point of view, there is not a clear association between the authors’ sections (2.1 ,2.2) and the biophysical theory of aforementioned works that have been applied successfully in numerous datasets and applications. In particular, I am completely lost when authors describing “combine (2.9) with (2.5), the output of the optimization procedure, and the reference (2.10) allows us to compute the logarithm of the dissociation constant of RBP-RNA binding …” in section 2.2 because I do not understand the reason for combining these two equations. After going through sections 3.1 and 3.2, I feel that the proposed method is actually in analogy to k-mer motif enrichment tests such as a paper in Ghandi M et.al. 2014 (Ref 6)
2. The manuscript describes motif prediction in RNA-binding proteins, but all of the figures are showing DNA binding motifs where T shall be replaced by U in the RNA sequence. It is very weird that the paper describes RNA-binding proteins but all sequence logo are DNA binding motifs. Authors have to correct these errors in all sequence logo figures. As we all know methods for motif finding in either DNA or RNA sequences are almost the same, but the prediction results (e.g., DNA-binding motif, RNA-binding motif) are not the same in biology.

3)  I am not able to compile and run cpp code that authors provided in the github, nor can I find demo input data to reproduce their results. Authors need to provide compiled cpp binary code (e..g,, Linux and MAC version) as well as relevant demo data for readers to reproduce some of their predictions in the manuscript.

Is the rationale for developing the new method (or application) clearly explained?

No

Is the description of the method technically sound?

No

Are the conclusions about the method and its performance adequately supported by the findings presented in the article?

No

If any results are presented, are all the source data underlying the results available to ensure full reproducibility?

No

Are sufficient details provided to allow replication of the method development and its use by others?

No

Reviewer Expertise:

Theoretical Physics, Computational biology, Bioinformatics, Applied Mathematics, Algorithm

I confirm that I have read this submission and believe that I have an appropriate level of expertise to state that I do not consider it to be of an acceptable scientific standard, for reasons outlined above.

## References

[1] : Selection of DNA binding sites by regulatory proteins. Statistical-mechanical theory and application to operators and promoters. *J Mol Biol* .1987;193(4) : 10.1016/0022-2836(87)90354-8 723-50 10.1016/0022-2836(87)90354-8 3612791

[2] : A biophysical approach to transcription factor binding site discovery. *Genome Res* .2003;13(11) : 10.1101/gr.1271603 2381-90 10.1101/gr.1271603 14597652 PMC403756

[3] : Statistical mechanical modeling of genome-wide transcription factor occupancy data by MatrixREDUCE. *Bioinformatics* .2006;22(14) : 10.1093/bioinformatics/btl223 e141-9 10.1093/bioinformatics/btl223 16873464

[4] : BayesPI - a new model to study protein-DNA interactions: a case study of condition-specific protein binding parameters for Yeast transcription factors. *BMC Bioinformatics* .2009;10: 10.1186/1471-2105-10-345 345 10.1186/1471-2105-10-345 19857274 PMC2771022

[5] : Identifying functional regulatory mutation blocks by integrating genome sequencing and transcriptome data. *iScience* .2023;26(8) : 10.1016/j.isci.2023.107266 107266 10.1016/j.isci.2023.107266 37520692 PMC10371843

[6] : Enhanced regulatory sequence prediction using gapped k-mer features. *PLoS Comput Biol* .2014;10(7) : 10.1371/journal.pcbi.1003711 e1003711 10.1371/journal.pcbi.1003711 25033408 PMC4102394

# Reviewer report: Reviewer response for version 2

**DOI:** 10.5256/f1000research.165610.r284354

**Published:** 2024-07-05

**Authors:** Johannes Söding

**Affiliations:** 1 Campus-Institut Data Science (CIDAS), Georg-August-Universitat Gottingen, Göttingen, Lower Saxony, Germany; 2 Quantitative and Computational Biology, Max Planck Institute for Multidisciplinary Sciences, Göttingen, Germany

I am glad to see that the method appears to work well after the adjustment to the background model.

 1. What other methods or software tools exist that can analyze Bind’n’seq data?

 2. How does your tool compare (quantitatively) with others to analyze Bind’n’seq data?

 Minor comments:

 3. Equation (2.4):  P(IG|S,c,M,E_0)  = … =>  P(S |  bound, c, M, E₀) =

 As a simple general rule, when you sum over all possible outcomes of all variables on the left of the conditioning bar |, you have to get 1. Here, summing over all possible sequences S in your library gives 1, thanks to the normalization in the denominator from Bayes’ rule.

 Same notation in the text a few lines below the equation.

 4. A bit below: P(S) = f_s is *not* a likelihood but a prior probability.

 5. In the conclusion, you wrote” In addition, it is possible that the binding sites of these proteins are not contiguous, linear motifs, but rather contain variable length spacers of form structures such as G-quadruplexes.” You could cite these manuscript here:

 (Jolma A, et al., 2020 [Ref 1]), ( Sohrabi-Jahromi S, 2021 [Ref 2])

Is the rationale for developing the new method (or application) clearly explained?

Yes

Is the description of the method technically sound?

Yes

Are the conclusions about the method and its performance adequately supported by the findings presented in the article?

No

If any results are presented, are all the source data underlying the results available to ensure full reproducibility?

Yes

Are sufficient details provided to allow replication of the method development and its use by others?

No

Reviewer Expertise:

Biophysics, statistical modeling

I confirm that I have read this submission and believe that I have an appropriate level of expertise to confirm that it is of an acceptable scientific standard, however I have significant reservations, as outlined above.

## References

[1] : Binding specificities of human RNA-binding proteins toward structured and linear RNA sequences. *Genome Res* .2020;30(7) : 10.1101/gr.258848.119 962-973 10.1101/gr.258848.119 32703884 PMC7397871

[2] : Thermodynamic modeling reveals widespread multivalent binding by RNA-binding proteins. *Bioinformatics* .2021;37(Suppl_1) : 10.1093/bioinformatics/btab300 i308-i316 10.1093/bioinformatics/btab300 34252974 PMC8275352

# Reviewer report: Reviewer response for version 1

**DOI:** 10.5256/f1000research.148267.r215390

**Published:** 2023-11-22

**Authors:** Jun Zhang

**Affiliations:** 1 Texas Tech University Health Science Center El Paso (TTUHSCEP), El Paso, USA

The manuscript presents a thermodynamic model for scrutinizing RNA motifs associated with RNA-binding proteins, utilizing position-specific weight matrices. Although the method effectively characterizes the RNA-binding specificity of RBFOX2, its application to other RNA-binding proteins, such as CELF1, HNRNPD, and HNRNPK, proves unsuccessful. The manuscript, categorized as a method paper, lacks comprehensive details about the model's development and fails to adequately address why the method specifically succeeds for RBFOX2.

 Key areas of improvement are outlined below:

1. **Unclear Definition of Parameter PIP:**
   
  - The manuscript lacks a clear definition of the parameter PIP. Providing a concise explanation will enhance reader understanding and facilitate the application of the method.
2. **Determination of Parameter c and its Relation to Protein Concentrations:**
   
  - Clarify how the parameter c is determined and elaborate on its relationship with the concentrations of RNA-binding proteins. A more in-depth explanation will contribute to the method's transparency.
3. **Thermodynamic Model Explanation:**
   
  - Offer a more detailed explanation of the thermodynamic model to eliminate the necessity for readers to consult the original reference. This will enhance accessibility and comprehension for a wider audience.
4. **Derivation of Equation 2.1:**
   
  - Clearly outline the derivation of Equation 2.1 to provide readers with insights into the model's foundational principles. This will aid in understanding the method's inner workings.
5. **Computation Time Information:**
   
  - Include information about the expected computation time, as this is crucial for users assessing the feasibility of implementing the method in their own studies.
6. **Evidence for Data Quality Variation:**
   
  - While the manuscript attributes the method's failure for CELF1, HNRNPD, and HNRNPK to lower data quality, provide concrete evidence supporting this claim. Offer a detailed analysis of the data quality disparities between RBFOX2 and the other proteins.
7. **Consideration of Variable Spacer Length in RNA-Binding Proteins:**
  - Acknowledge the fact that many RNA-binding proteins, including CELF1, HNRNPD, and HNRNPK, bind to RNA with variable spacer lengths. The manuscript should discuss why the PWM method, assuming a fixed RNA motif length, may be inadequate for proteins with multiple RNA-binding domains connected by long linkers. This acknowledgment will help users understand the method's limitations and guide appropriate applications.

In conclusion, addressing these points will significantly enhance the manuscript's clarity, transparency, and utility, providing a more comprehensive understanding of the developed method and its limitations.

Is the rationale for developing the new method (or application) clearly explained?

Partly

Is the description of the method technically sound?

Yes

Are the conclusions about the method and its performance adequately supported by the findings presented in the article?

Partly

If any results are presented, are all the source data underlying the results available to ensure full reproducibility?

No source data required

Are sufficient details provided to allow replication of the method development and its use by others?

No

Reviewer Expertise:

RNA-binding protein, structural biology.

I confirm that I have read this submission and believe that I have an appropriate level of expertise to state that I do not consider it to be of an acceptable scientific standard, for reasons outlined above.

# Response

**Published:** 2024-07-22

**Authors:** niels schlusser

Dear Reviewer, 

 We apologize for the delay in submitting the answer to your valuable comments. The delay was caused by a technical issue with the web portal.

 Thank you for the insightful comments and questions related to our paper, which we have

 taken into careful consideration in revising our manuscript. We believe the manuscript is

 much improved as a result. Below we comment on the most significant changes.

 As a result of the revised terminology and calculation of frequency priors f_S, some of the

 comments have become superfluous. Nevertheless, the main comments were addressed as

 follows:

1. We clarified the definition of P_IP in and around Eq. (2.4), and the notation has changed.
2. Around Eq. (2.1), we elaborated on the role of the ratio of concentrations of bound and unbound RBP c.
3. We explained the derivation of the thermodynamic model in greater detail (c.f. above comments) so that it does not require the reader to consult the cited reference of [1].
4. We also gave more detail around Eq. (2.1) (c.f. item 2).
5. Information about the computation time is given in the first paragraph of Sec. 4 ”results”.
6. Regarding the evidence for data quality variation: we have applied the model to the entire set of RBPs assayed by RNA Bind’n Seq and, with the revised prior calculation we recovered the consensus motifs for 48 out of 82 RBPs. While in response to the comments of reviewer #1 we also tried initialization with the consensus motif/enriched kmers, the results for the 34 proteins for which random initialization did not lead to the recovery of meaningful, non-poly(A) motif, have not improved. Thus, our model is, in principle, able to recover the expected binding motifs.
7. The reviewer suggested that the PWM model may be inadequate for many RBPs that bind to discontinuous motifs. While a large body of prior work uses the PWM representation of RBP binding sites, there are known example where structure plays a role. How to appropriately represent structured binding sites is an open problem that we think is beyond the scope of our work. Nevertheless, we added a comment about this possibility explaining some of our results in the discussion.

We hope we have adequately responded to your comments.

 Yours sincerely,

 The authors

 References

 [1] S. Omidi, M. Zavolan, M. Pachkov, J. Breda, S. Berger, and E. van Nimwegen, Automated incor-

 poration of pairwise dependency in transcription factor binding site prediction using dinucleotide

 weight tensors, PLOS Computational Biology 13 (2017) 1.

# Reviewer report: Reviewer response for version 1

**DOI:** 10.5256/f1000research.148267.r192424

**Published:** 2023-08-31

**Authors:** Johannes Söding

**Affiliations:** 1 Campus-Institut Data Science (CIDAS), Georg-August-Universitat Gottingen, Göttingen, Lower Saxony, Germany; 2 Quantitative and Computational Biology, Max Planck Institute for Multidisciplinary Sciences, Göttingen, Germany

The manuscript describes a method to learn  position-weight matrices (PWM) to describe the sequence specific binding of an RBP given Bind'n'seq data for this RBP. The method is based on a biophysically motivated, statistical model for the likelihood of Bind'n'seq data given the PWM to describe the sequence specific binding of the RBP to the library DNA oligomers. The model parameters (PWM and non-specific binding energy E₀) are learned using an expectation maximization algorithm. The method is applied to Bind'n'seq data for 9 RBPs. For some RBPs correct PWMs are retrieved. For others, the most likely PWM does not describe the known binding motif but is rather an unrelated, low-complexity motif that appears to reflect simple sequence biases in the experiment.  

 The statistical model is very sound and the manuscript is well written. The results are disappointing, in particular since it is not clear whether the negative results for a good part of the data sets are due to the inadequacy of the statistical model or some bug in the implementation. 

 1. My guess is the following. The method uses a Markov model of order d to model the prior probability P(S) = f_S for a sequence S to be part of the background library (before enrichment). It was found that "the better the prediction of likelihood of sequences in the foreground samples." This led the authors to set d=14. A Markov model of order d=14 has 3*4^28 ~ 10^9 parameters, around the same number as 14-mers in the entire sequencing library of around 10^7 reds. This means the Markov model is hopelessly over-parameterized and the reason that the likelihood was increasing for increasing d is simply that the overlap between the (d+1)-mers in the background and foreground libraries decreases with increasing d. A reasonable choice of d is around 4, certainly not more than 8. The overfitting of the pior probability model could explain the many weird local optima observed by the authors, since it adds enormous noise to the sequence space such that a single mutation can change the f_s dramatically, resulting in many spurious PWM  minima.

 2. The notation in equations (2.1) to (2.4) is quite "unstatistical" and confusing. The way (2.1) and  (2.3) are written, the left hand side would need to sum to one when summing over all sequences s or S, respectively. This is of course not the case. What the authors rather meant to write on the left hand side of eq (2.1) is P(bound | s,c,M,E₀), with a variable  bound in {0,1} indicating whether the sequence on the right side of the conditioning is bound by the RBR or not. The left hand side of eq. (2.3) should read P(bound | S,c,M,E₀).

 With this corrected notation and using f_S = P(S) for the prior probability of finding a sequence S in the background library (with ∑_S P(S) = 1), equation (2.4) turns into the correct Bayes theorem,

    P(S | bound,c,M,E₀) = P(bound | S,c,M,E₀)  P(S) /  ∑_σ  P(bound | σ,c,M,E₀)  P(σ) .

 Please correct the text accordingly: 

 "(2.4) is essentially a formulation of Bayes’ theorem with conditional probability P_b(S|c,M,E₀) of having a read S bound by an RBP, the likelihood of finding a read S in the pool washed over the RBP, fS, and an overall normalization (denominator)." 

 =>

 "(2.4) is essentially a formulation of Bayes’ theorem with the likelihood P(bound | S,c,M,E₀) of having a read S bound by an RBP, the likelihood of finding a read S in the pool washed over the RBP, P(S)=f_S, and an overall normalization in the denominator." 

 3. To improve the search for the global optimum, the authors could compute the most highly enriched 6-mers and start their PWM optimization from a soft version of each of the top 20 or so 6-mers. 

 4. Please derive the EM update equations transparently.

Is the rationale for developing the new method (or application) clearly explained?

Yes

Is the description of the method technically sound?

Yes

Are the conclusions about the method and its performance adequately supported by the findings presented in the article?

No

If any results are presented, are all the source data underlying the results available to ensure full reproducibility?

Yes

Are sufficient details provided to allow replication of the method development and its use by others?

No

Reviewer Expertise:

Biophysics, statistical modeling

I confirm that I have read this submission and believe that I have an appropriate level of expertise to confirm that it is of an acceptable scientific standard, however I have significant reservations, as outlined above.

# Response

**Published:** 2024-04-26

**Authors:** niels schlusser

Dear reviewer,

 Thank you for the insightful comments and questions related to our paper, which we have

 taken into careful consideration in revising our manuscript. We believe the manuscript is

 much improved as a result. Below we comment on the most significant changes.

 Addressing your feedback:

 1. You suggested that we do not over-parameterize the background model, but

 rather use a Markov model of order 4−8. We followed this suggestion, setting d = 4 and

 adapting the normalization of frequency priors accordingly. While implementing this

 suggestion we also noticed a mistake in the initial implementation of the normalization,

 which likely contributed to higher order background giving improved results initially.

 As a result of these changes we found non-trivial motifs for a larger proportion of the

 RBPs, which led us to apply the model to all RBP RNA Bind’seq datasets in ENCODE

 (111 in total).

 2. You also pointed out some sloppiness in our initial notation, which we have

 now corrected, along with the text referring to the respective equation (2.4).

 3. We did try various ways of initializing the PWM from enriched kmers and consensus

 motifs, but the number of cases where this led to the convergence to the expected motif,

 while the random initialization did not, was very small.

 4. We elaborated more on the derivation of the EM update equations, in particular, we

 give the derivative whose root is calculated.

 We hope we have adequately responded to your comments.

 Yours sincerely,

 Mihaela Zavolan and Niels Schlusser
